# Recent range expansion in Australian hummock grasses (*Triodia*) inferred using genotyping-by-sequencing

**DOI:** 10.1093/aobpla/plz017

**Published:** 2019-03-28

**Authors:** Benjamin M Anderson, Kevin R Thiele, Pauline F Grierson, Siegfried L Krauss, Paul G Nevill, Ian D Small, Xiao Zhong, Matthew D Barrett

**Affiliations:** 1 School of Biological Sciences, The University of Western Australia, Crawley, Western Australia, Australia; 2 Kings Park and Botanic Garden, Botanic Gardens and Parks Authority, Kings Park, Western Australia, Australia; 3 Australian Research Council Centre for Mine Site Restoration, Curtin University, Bentley, Western Australia, Australia; 4 Australian Research Council Centre of Excellence in Plant Energy Biology, School of Molecular Sciences, The University of Western Australia, Crawley, Western Australia, Australia

**Keywords:** Arid zone, Australia, biogeography, chloroplast, divergence dating, genotyping-by-sequencing, Poaceae, range expansion, SNPs, *Triodia basedowii* species complex

## Abstract

The Australian arid zone (AAZ) has undergone aridification and the formation of vast sandy deserts since the mid-Miocene. Studies on AAZ organisms, particularly animals, have shown patterns of mesic ancestry, persistence in rocky refugia and range expansions in arid lineages. There has been limited molecular investigation of plants in the AAZ, particularly of taxa that arrived in Australia after the onset of aridification. Here we investigate populations of the widespread AAZ grass *Triodia basedowii* to determine whether there is evidence for a recent range expansion, and if so, its source and direction. We also undertake a dating analysis for the species complex to which *T. basedowii* belongs, in order to place its diversification in relation to changes in AAZ climate and landscapes. We analyse a genomic single nucleotide polymorphism data set from 17 populations of *T. basedowii* in a recently developed approach for detecting the signal and likely origin of a range expansion. We also use alignments from existing and newly sequenced plastomes from across Poaceae for analysis in BEAST to construct fossil-calibrated phylogenies. Across a range of sampling parameters and outgroups, we detected a consistent signal of westward expansion for *T. basedowii*, originating in central or eastern Australia. Divergence time estimation indicates that *Triodia* began to diversify in the late Miocene (crown 7.0–8.8 million years (Ma)), and the *T. basedowii* complex began to radiate during the Pleistocene (crown 1.4–2.0 Ma). This evidence for range expansion in an arid-adapted plant is consistent with similar patterns in AAZ animals and likely reflects a general response to the opening of new habitat during aridification. Radiation of the *T. basedowii* complex through the Pleistocene has been associated with preferences for different substrates, providing an explanation why only one lineage is widespread across sandy deserts.

## Teaser

Much of Australia is covered by an arid zone that has developed over the last 15 million years (Ma). The unique flora that inhabits that arid zone includes plants that have responded differently (expanding or contracting their ranges) to climate and landscape changes. Here we show that one widespread desert grass, *Triodia basedowii*, has recently (within the last 2 Ma) expanded across the arid zone. Surprisingly, we find evidence of an expansion origin in central or eastern Australia, while the bulk of the diversity in the group to which *T. basedowii* belongs exists in western Australia.

## Introduction

Large-scale environmental changes such as aridification have shaped and continue to influence the evolution and composition of biomes. As conditions change, organisms may persist in some parts of their ranges, become extinct in others, and/or may migrate into newly available or previously inaccessible ecologically favourable regions. Understanding the drivers behind range shifts and biome assembly can be aided by revealing current patterns of genetic diversity in taxa occupying regions known to have been influenced by geologically recent large-scale environmental changes.

The Australian arid zone (AAZ) provides an excellent location for exploring the influence of continent-scale aridification on biome assembly (reviewed in Byrne *et al.* 2008). Since the mid-Miocene *c*. 16 million years (Ma) ago, climate in inland Australia has shifted from predominantly mesic (Martin 2006) to current arid conditions (e.g. average annual precipitation less than ~500 mm per year). Concurrently, there has been extensive landform change (Fujioka and Chappell 2010), leading to the formation of new habitats for AAZ biota. From the onset of the Pleistocene *c*. 2.6 Ma (Walker *et al.* 2012), global glacial cycles began to produce alternately warm/wet and cool/dry conditions in Australia (Williams 1984), superimposed on a trend of global cooling (Zachos *et al.* 2001). The onset of cooling and glacial cycles correspond to the earliest evidence for stony deserts in Australia *c*. 2–4 Ma (Fujioka *et al.* 2005). Beginning roughly 0.8–1.2 Ma, the glacial cycles increased in amplitude and decreased in frequency (Pisias and Moore 1981; Mudelsee and Stattegger 1997; Clark *et al.* 1999); this climatic transition coincides with the earliest evidence for sandy dunefields in central Australia *c*. 1 Ma (Fujioka *et al.* 2009), which suggests heightened aridity. Some regional variation is evident within this overall trend, with, for example, drier conditions in north-western Australia from as early as the mid-Miocene (Groeneveld *et al.* 2017) followed by the humid interlude *c*. 5.5–3.3 Ma before a return to arid conditions by 2.4 Ma (Christensen *et al.* 2017).

The assembly of the AAZ biome included both taxa that descended from mesic-adapted groups present in Australia before the onset of aridity and taxa that descended from more recent and likely arid-adapted dispersals (see Crisp and Cook 2013). Often, descendents of mesic groups are restricted to range systems or putative environmental/substrate refugia, e.g. geckos (Fujita *et al.* 2010; Oliver *et al.* 2010, 2014; Pepper *et al.* 2011a, b), pebble-mimic dragons (Shoo *et al.* 2008), blindsnakes (Marin *et al.* 2013), grasshoppers (Kearney and Blacket 2008) and the conifer *Callitris* (Sakaguchi *et al.* 2013). In other cases, descendents of mesic groups have presumably adapted to desert conditions and show evidence of recent range expansions in sandy deserts surrounding the rocky range systems (Strasburg and Kearney 2005; Kearney and Blacket 2008; Fujita *et al.* 2010; Pepper *et al.* 2011b). Decendents of more recent dispersals tend to show a pattern of radiation into the expanding arid zone, with plant examples including chenopods (Shepherd *et al.* 2004; Kadereit and Freitag 2011), *Lepidium* (Mummenhoff *et al.* 2004), *Triodia* (Toon *et al.* 2015) and probably *Ptilotus* (Hammer *et al.* 2015). Animal examples include rodents (Rowe *et al.* 2008) and elapid snakes (Keogh *et al.* 1998), although those radiations occurred across the continent and presumably did not involve pre-adaptation of the entire group to arid conditions.

An outstanding knowledge gap surrounds how AAZ plants, especially recent dispersals with arid ancestry, have responded to climatic and landscape changes since the mid-Miocene and during glacial cycles. Arid-adapted lineages might be expected to respond favourably to cool/dry periods as in, e.g., the Chilean Atacama desert (Ossa *et al.* 2013). Evidence to date, however, suggests that cool/dry periods in Australia may have been difficult for the majority of the flora, including the arid-adapted lineages, as evidenced by declines in C_4_ grasses around the last glacial maximum in central Australia (Johnson *et al.* 1999; Smith 2009) and from dust and pollen records indicating reduced vegetation cover during glacial maxima (Hesse *et al.* 2004; Martin 2006). Here we investigate the response of a group of arid-adapted hummock grasses from the AAZ, the *Triodia basedowii* species complex.

Perennial grasses in the genus *Triodia* are iconic Australian plants and dominant components of hummock grasslands, which cover >18 % of the continent (Department of the Environment and Water Resources 2007). These hummock grasses in general show a wide ecological adaptability and are distributed across some of the driest parts of the continent, extending into tropical savannahs in northern Australia and semi-arid temperate regions in the south (Lazarides 1997). They are ecologically important and provide food and/or habitat for a variety of animals (Ealey *et al.* 1965; Dawson and Bennett 1978; Kitchener *et al.* 1983; Losos 1988; Morton and James 1988; Daly *et al.* 2008; Christidis *et al.* 2010; Laver *et al.* 2017). *Triodia* is a member of the subfamily Chloridoideae (Peterson *et al.* 2010), a group of C_4_ grasses thought to have initially diversified in dry habitats in Africa in the Oligocene (Bouchenak-Khelladi *et al.* 2010). The closest relatives of *Triodia*, variably recovered as *Aeluropus*, *Orinus* or *Cleistogenes* (Peterson *et al.* 2010; Grass Phylogeny Working Group II 2012), occur in dry or sandy environments from the Mediterranean to Japan (Clayton and Renvoize 1986; Watson and Dallwitz 1992). Ancestors of *Triodia* are thought to have arrived in Australia *c*. 24–14 Ma (Toon *et al.* 2015), probably already at least partly adapted to arid conditions.

The *T. basedowii* species complex is found across much of the central AAZ, with the bulk of its diversity in the Pilbara region of north-western Australia (Anderson *et al.* 2016). Following a recent taxonomic revision (Anderson *et al.* 2017a), the complex includes nine species: *T. basedowii*, *T. lanigera*, *T. birriliburu*, *T. chichesterensis*, *T. glabra*, *T. mallota*, *T. nana*, *T. scintillans* and *T. vanleeuwenii*. Of these nine species, only *T. basedowii* is widespread across sandy habitats of the AAZ, while many of the others are restricted to discrete geographic areas and substrates. Given the high lineage richness in the Pilbara (Anderson *et al.* 2016), it might be expected that *T. basedowii* expanded its range eastward from there, but this remains to be tested.

In this study, we examine evidence for range expansion in *T. basedowii* to identify a source area using novel analyses of a previously published genomic single nucleotide polymorphisms (SNPs) data set (Anderson *et al.* 2017b). In addition, we put the diversification of the *T. basedowii* complex in a temporal context using new and existing chloroplast genomic sequences to relate to climate and landscape changes in the AAZ.

## Materials and Methods

### Range expansion

To evaluate range expansion in *T. basedowii*, we used genomic SNPs obtained from a previously published genotyping-by-sequencing data set (Anderson *et al.* 2017b). We included 36 samples of *T. basedowii* from 17 populations across its range **[see****Supporting Information—Table S1****]**, along with three samples of *T. birriliburu*, four of *T. nana* and five of *T. glabra* to be used alternately as outgroups for determining ancestral and derived SNP states. Genomic SNP data sets were generated following a modification of a genotyping-by-sequencing approach (Elshire *et al.* 2011), using primers and barcodes from J. Borevitz (Grabowski *et al.* 2014). Paired-end reads were assembled using PyRAD v. 3.0.6 (Eaton 2014) based on optimal clustering thresholds determined in Anderson *et al.* (2017b). The generated data sets consisted of target *T. basedowii* samples as well as samples from one of the outgroups, with SNPs retained only when they were present in at least four samples. SNPs were obtained from two sets of loci: ‘assembled’ loci that had been generated from overlapping reads merged by PEAR (Zhang *et al.* 2014) and ‘unassembled’ loci that had not, i.e. shorter and longer sequenced fragments with greater and lesser read depth, respectively (see Anderson *et al.* 2017b). We used custom Python (Python Software Foundation 2016) scripts to select a single SNP per locus either (i) randomly or (ii) with a bias towards biallelic SNPs with multiple copies of the rare allele. We used a custom R v. 3.2.5 (R Development Core Team 2015) script to filter the resulting data sets to keep only SNPs present in at least one outgroup sample and to format them for the range expansion analyses.

The range expansion analyses implemented here use a measure developed by Peter and Slatkin (2013, 2015) based on the difference in derived allele frequencies for biallelic SNPs between two populations. If a source population extends its range through a series of founder events, it is expected that populations further from the origin of the expansion will have experienced more genetic drift, producing clines in the frequencies of neutral alleles (as alleles are lost with each subsequent founder event), and leading to populations further away from the origin having higher frequencies of derived alleles (Peter and Slatkin 2013). Peter and Slatkin (2013) observed that the measure increased linearly with distance from the origin of an expansion, so it could be used with a time difference of arrival method (Gustafsson and Gunnarsson 2003) to detect the most likely location of the origin of an expansion for a set of populations. The approach has been used to infer origins of expansions in a group of tropical skinks (Potter *et al.* 2016) and to support similar inferences for monarch butterflies (Zhan *et al.* 2014), coralsnakes (Streicher *et al.* 2016), hares (Marques *et al.* 2017) and zebras (Pedersen *et al.* 2018). Dr Peter kindly provided scripts implementing these analyses, which we adjusted to fit our data and geographic area of interest. The scripts use the R packages ‘geosphere’ v. 1.5-1 (Hijmans 2015), ‘sp’ v. 1.2-2 (Pebesma and Bivand 2005), ‘rworldmap’ v. 1.3-6 (South 2011), ‘maps’ v. 3.1.0 (Becker *et al.* 2016) and ‘mapproj’ v. 1.2-4 (McIlroy *et al.* 2015).

Subsets of the SNP data sets were run through the expansion scripts to assess consistency of any signal of expansion depending on choice of outgroup, inclusion or exclusion of polyploids, numbers of individuals per population and presence of geographic structure. Separate analyses were run for these variations using each of the three outgroup taxa. Some populations of *T. basedowii* are tetraploid and have a slightly higher individual heterozygosity (Anderson *et al.* 2017b). Given that higher heterozygosity in polyploids could affect a signal based on allele frequencies, we ran analyses including and excluding tetraploid populations. Population sampling was uneven, so we ran the scripts by either (i) randomly choosing a single individual per population for comparison or (ii) allowing the scripts to downsample larger populations. The expansion scripts assume a single origin; in the case of multiple suspected origins, Peter and Slatkin (2013) recommend estimating which samples are likely to have come from each origin (e.g. using geographic structure) and then applying their method to each group of samples separately. We assessed geographic structure in *T. basedowii* using genomic SNPs in a principal components analysis (PCoA; ‘cmdscale’ function in R) based on Euclidean distances (‘dist’ function in R) between samples. The 8663 SNPs used in the PCoA were obtained using *T. birriliburu* as an outgroup, and were randomly selected from ‘assembled’ loci. Clusters of samples apparent in the PCoA were run separately in the range expansion analyses, in addition to running all samples together. The accuracy of origin detection is reduced and should be interpreted cautiously if the origin is near or beyond the edge of the sampled area (Peter and Slatkin 2013). Initial analyses sometimes recovered the origin at the edge of the area bounded by our sampling, so we broadened the geographic area for detecting the origin.

### Diversification timing

We newly sequenced and assembled 28 Poaceae plastomes and downloaded 26 more from GenBank **[see****Supporting Information—Table S2****]** to conduct fossil-calibrated dating analyses across the grasses (data set 1) and within the Chloridoideae (data set 2) using BEAST v. 2.4.6 (Bouckaert *et al.* 2014). We ran multiple analyses to assess the impact of calibration, model choice and alignment length. We evaluated results based on two placements of controversial early grass fossils (Prasad *et al.* 2005, 2011; see Christin *et al.* 2014). Clock models included the relaxed uncorrelated log-normal (UCLN; Drummond *et al.* 2006) and a random local clocks model (RLC; Drummond and Suchard 2010). Further details of the sequencing and analyses are included in **Supporting Information**.

## Results

### Range expansion

**Figure 1.**
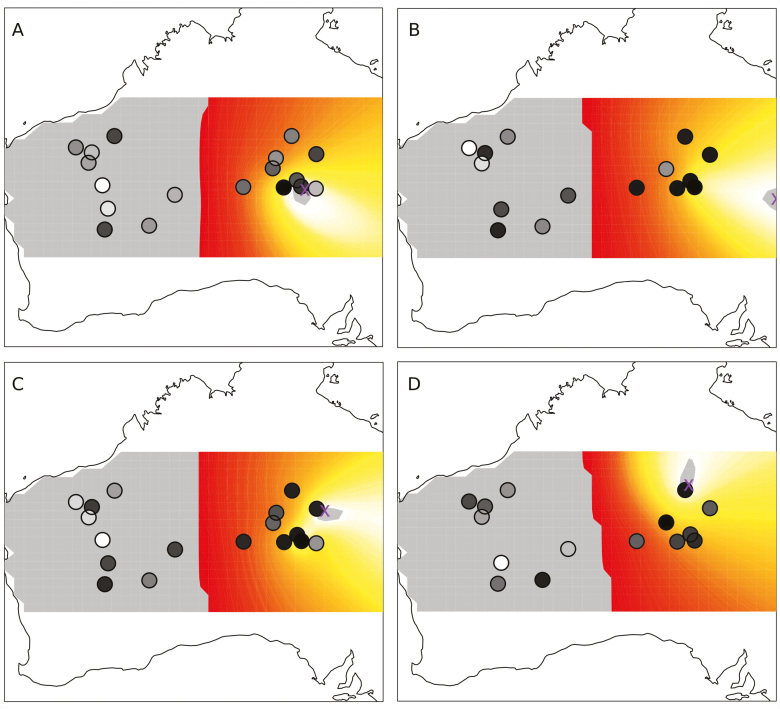
Some of the results of range expansion analyses for *Triodia basedowii* using different genomic SNP subsets. Ancestral states of SNPs were determined using *T. birriliburu* (A, B), *T. nana* (C) or *T. glabra* (D) as outgroups. Loci sets were assembled (A, C), unassembled (D) or a combination of the two (B). SNPs were chosen randomly (A, D) or with a bias towards SNPs with multiple copies of the rare allele (B, C). Population sampling was one per population (A), one per population excluding tetraploids (D), all samples (C) and all samples excluding tetraploids (B). Panels show heat maps and probable locations of the origin of range expansion (purple ‘X’). Populations are indicated with circles, where darker shades show higher heterozygosity.

Across multiple analyses we recovered a consistent signal of westward expansion for *T. basedowii* (a subset is shown in Fig. 1; see also **Supporting Information—Table S3**), regardless of outgroup choice, inclusion or exclusion of polyploids, or sampling of genomic SNPs. The origin was variously resolved in central or eastern Australia, sometimes outside the current known range of *T. basedowii*. While the precise location of the inferred origin was not consistent, the analyses only recovered the origin in the eastern portion of the study area.

**Figure 2.**
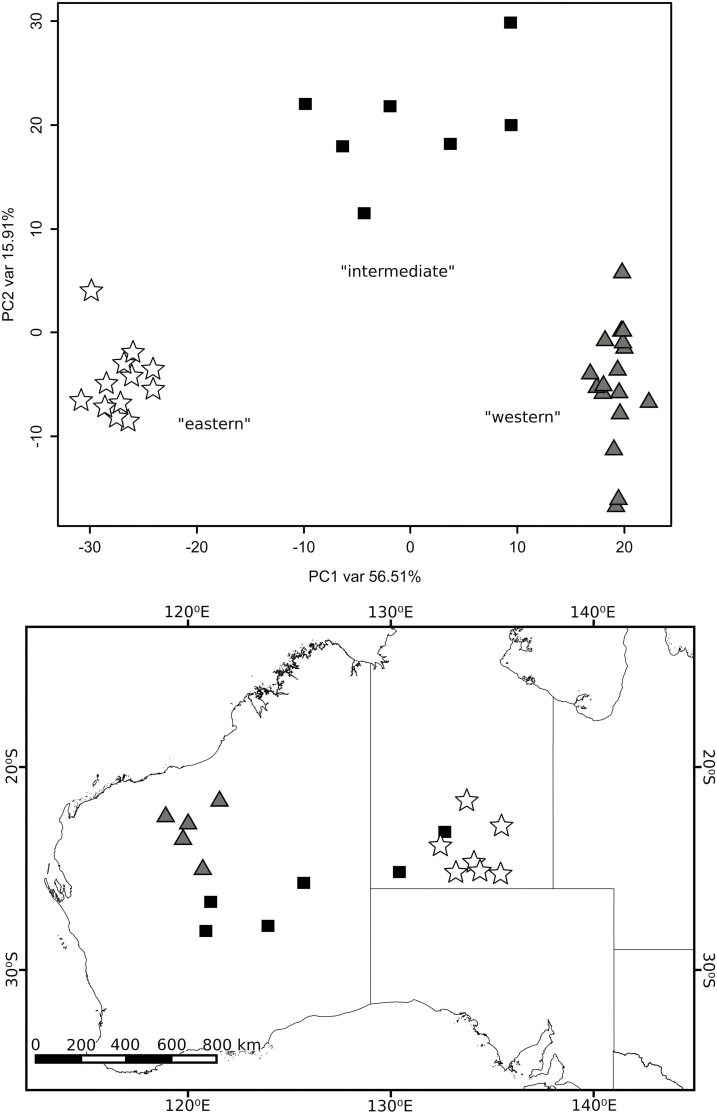
Principal components analysis (PCoA) of genomic SNPs from individual plants of *Triodia basedowii*. Population localities are shown on the map with symbols reflecting their grouping in the PCoA.

To evaluate the possibility of multiple expansion origins, we examined geographic structure within *T. basedowii* in a PCoA (Fig. 2). There was strong evidence for geographic clustering, with three clearly distinct groups of samples: a ‘western’ group focused near the Pilbara, an ‘eastern’ group in central Australia and an ‘intermediate’ group distributed between these two. Running the range expansion analyses on the groups separately did not recover significant (*P* < 0.01) signals of expansion compared to isolation by distance **[see****Supporting Information—Table S3****]**, with a single exception for the eastern group. In almost all analyses, significant signals of expansion were only detected for all samples combined.

### Diversification timing

**Figure 3.**
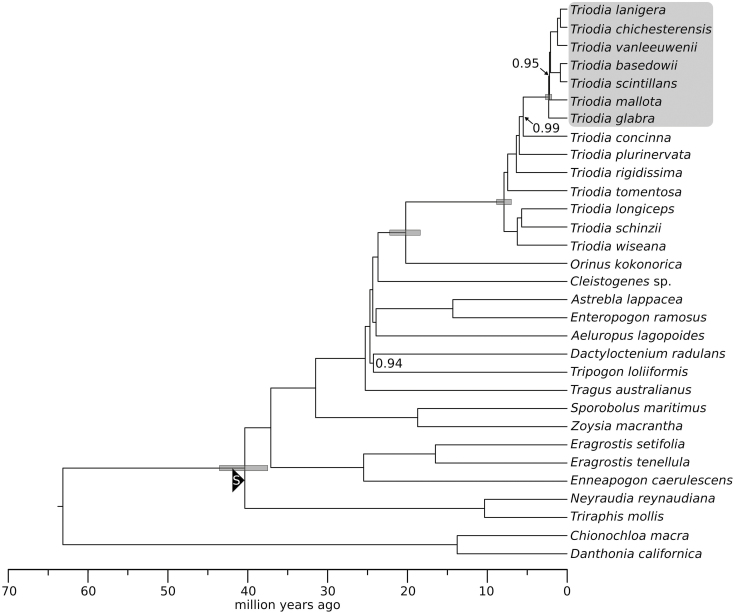
Chronogram from the BEAST analysis of data set 2, comprising chloroplast alignments with a focus on Chloridoideae and *Triodia*, with two outgroup samples from Danthonioideae. The secondary calibration from the analysis of data set 1 is indicated by the ‘S’ in a black triangle. Node bars for selected nodes of interest are 95 % highest posterior density intervals. Node support values are posterior probabilities and are shown for values <1.00. The *Triodia basedowii* complex is shaded at the top right.

Phylogenetic relationships within Poaceae were recovered **[see****Supporting Information—Figs S1–S4****]** consistent with current understanding of grass evolution (Grass Phylogeny Working Group II 2012; Soreng *et al.* 2015) and node ages for most major splits (Table 1; **see****Supporting Information—Fig. S5**) were comparable to estimates from recent studies (Prasad *et al.* 2011; Christin *et al.* 2014; Burke *et al.* 2016). Estimated node ages within Chloridoideae (Table 2; Fig. 3) indicate that *Triodia* began to diversify in the late Miocene (7.9 Ma; 7.0–8.8 Ma 95 % highest posterior density interval [HPD]), and that the *T. basedowii* complex began to radiate in the Pleistocene (2.3 Ma; 1.9–2.7 Ma 95 % HPD). Alternative placement of the controversial fossils had minimal impact **[see****Supporting Information—Table S6****]** on the crown age of the complex (1.7 Ma; 1.4–2.0 Ma 95 % HPD), and still indicated a Pleistocene radiation.

**Table 1.** Node ages (Ma) from analysis of data set 1 compared to previous molecular dating of the grasses. Node ages from Prasad *et al.* (2011) are with placement of the phytoliths at stem Oryzeae (their H1), while those from Christin *et al.* (2014) are based on their BEAST analysis of chloroplast data from across angiosperms, which included the placement of the phytoliths at stem Oryzeae. HPD is the highest posterior density interval.

Study	Current study		Prasad *et al.* (2011)		Christin *et al.* (2014)		Burke *et al.* (2016)	
Node	Age	95 % HPD	Age	95 % HPD	Age	95 % HPD	Age	95 % HPD
Crown Poaceae	123	119–125	121	95.9–149	88.5	80.9–97.8	106	99.5–110
Crown BOP + PACMAD	82.4	78.5–86.5	81.6	69.6–93.8	74.5	70.3–80	85.7	75.7–97.6
Crown Oryzoideae	65.6	65–66.6	67.1	56.9–77	68	67–70.8	72.9	66–87.9
Crown Bambusoideae	50	40.9–59	47.4	36.5–59.7	34.2	19.8–56.2	41.5	2.9–63.8
Crown Pooideae	60.1	55.1–65.2	57.8	48.2–67.6	59.9	51.4–68.5	62.9	50.1–75.7
Crown Chloridoideae	41.7	38.1–45.7	33.6	24.5–42.5	41.2	33.2–49	37.3	22.6–52.7

**Table 2.** Node ages (Ma) from analyses of data set 2. Node ages from Toon *et al.* (2015) are shown for comparison. HPD is the highest posterior density interval, RLC is a random local clocks model and UCLN is an uncorrelated log-normal clock model.

Node	Stem *Triodia*		Crown *Triodia*		Crown *T. basedowii* complex	
Analysis	Age	95 % HPD	Age	95 % HPD	Age	95 % HPD
RLC	20.2	18.4–22.2	7.89	6.98–8.82	2.29	1.91–2.70
UCLN	18.1	15.9–20.5	5.62	4.86–6.39	1.58	1.29–1.87
*matK* only	19.8	11.9–28.0	10.4	5.50–17.2	3.8	1.29–7.36
Toon *et al.* (2015)	20.9	17.9–23.5	14.7	11.4–18.3	4.58	2.60–6.86

## Discussion

### Expansion of *T. basedowii* across the sandy dunefields

Our results indicate that *T. basedowii* has undergone a recent east-to-west range expansion, with the origin most likely in central or eastern Australia. This finding augments an increasing number of studies indicating recent range expansions in AAZ lineages (e.g. Kearney and Blacket 2008; Fujita *et al.* 2010; Pepper *et al.* 2011b). While arid conditions in Australia have a history dating back to the Miocene, the more recent heightened aridity and formation of sandy dunefields *c*. 1 Ma (Fujioka *et al.* 2009) are likely to have created a widespread and relatively open niche onto which AAZ organisms with adaptations for sandy environments could expand. Geographic structuring in the *T. basedowii* complex (see Anderson *et al.* 2016) is strongly associated with substrate differences, and species now associated with rockier areas tend to grow poorly when cultivated in sand (e.g. *T. vanleeuwenii*; P. F. Grierson, University of Western Australia, unpubl. res.). Substrate has been implicated as a factor limiting the distributions of other AAZ organisms, principally as rocky refugial areas separated by inhospitable sandy habitat (e.g. Shoo *et al.* 2008; Oliver *et al.* 2014). We hypothesize that much of the AAZ flora with similar widespread distributions across sandy regions will show evidence of recent range expansion, and that elements of the AAZ flora with sand affinities will have lower lineage richness than their sister groups in rocky areas, given the relatively young age of the sandy deserts. Both these hypotheses require further investigation.

While the pairwise signal for expansion between populations varied across sampling schemes, the consistency of an east-to-west signal suggests that the Pilbara, at the western end of the range of the species, was not a source area for *T. basedowii* prior to its expansion across sandy dunefields. Previous work on the *T. basedowii* complex (Anderson *et al.* 2016) found no evidence for a refugium in central Australia, but was unable to look at diversity within *T. basedowii* populations. Based on the findings of the present study, we suggest that the Central Ranges or some other region of central Australia may have maintained populations of *T. basedowii* during the formation of the sandy dunefields and/or through Pleistocene glacial cycles. Our findings of a population expansion from eastern or central Australia also imply that Pilbara populations of *T. basedowii*, which are restricted to the sandy Fortescue River valley, are relatively recent incursions. These recent incursions may explain examples of hybridization with Pilbara species. One example of possible introgression involves *T. lanigera*, which based on genomic SNP data (Anderson *et al.* 2017b) is part of a different clade and closely related to *T*. *chichesterensis*, but which has an internal transcribed spacer (ITS) sequence highly similar to that of *T. basedowii* (Anderson *et al.* 2016). This pattern of ITS introgression in the absence of genomic mixing has previously been observed in a mixed population of *T. lanigera* and T. chichesterensis, the individuals of which shared ITS copies but were well differentiated across thousands of genomic SNPs (Anderson *et al.* 2017b).

The demographic history of *T. basedowii* is likely more complex than the single point source of expansion assumed by the model of Peter and Slatkin (2013), as some populations may have diverged prior to acting as sources for others. Across most sampling schemes, however, we failed to detect significant signals of expansion from individual geographic groups. The geographic structure in *T. basedowii* suggests a level of differentiation, perhaps due to periods of minimal genetic exchange following an initial expansion (possibly during glacial maxima) or through ongoing isolation by distance. Recently, a simulation study (He *et al.* 2017) has challenged the reliability of the analyses we used for detecting an origin when the underlying heterogeneity of the environment through time is not taken into account. This is a particular challenge for researchers working in the AAZ, as our knowledge of the extent of suitable habitat for AAZ species since the mid-Miocene is poor (e.g. the extent of sandy dunefields through the Pleistocene). In the case of *Triodia*, with its varied substrate preferences across species, it is an additional challenge to predict large-scale suitability when species turnover can be at a fine scale in the landscape. Our sampling of *T. basedowii* (36 samples across 17 locations) is low compared to many population genetics studies, but similar sampling has been used with this approach (e.g. Potter *et al.* 2016; Streicher *et al.* 2016), and limitations in statistical power from lower sampling of individuals are partly offset by the large number of markers in comparisons (~2000–7000; **see****Supporting Information—Table S3**; e.g. Willing *et al.* 2012). The variability in the location of the origin in our analyses suggests limitations of the method and/or the completeness of our genomic SNP sampling, as genotyping-by-sequencing data sets are characterized by high levels of missing data. While these challenges raise uncertainty as to the precise location of an origin, the finding of a consistent large-scale pattern of westward expansion remains robust.

### Diversification timing in the *T. basedowii* complex

Our dating analyses provide a new estimate for the timing of diversification in *Triodia* and the *T. basedowii* complex. We estimate that ancestors of *Triodia* diverged from their Asian relatives ~20 Ma, subsequently migrated to Australia and had begun to diversify by the late Miocene *c*. 8 Ma. Extant diversity in the *T. basedowii* complex arose from a crown radiation that began about 1.9–2.7 Ma in the Pleistocene and continued through glacial cycles and the climatic transition *c*. 1 Ma that led to the formation of sandy dunefields. This finding contrasts with patterns seen in some AAZ lineages that have a mesic ancestry, where species divergences often date to the Miocene, and Pleistocene glacial cycles appear to have affected phylogeographic structure rather than speciation (Byrne *et al.* 2008; e.g. Pepper *et al.* 2011b; Marin *et al.* 2013). A recent study by Toon *et al.* (2015) had limited sampling of the *T. basedowii* complex and indicated a somewhat older crown radiation in the Pliocene (see Table 2). Discrepancies between our dates and those of Toon *et al.* (2015) may be the result of data set size (chloroplast vs. ITS + *matK*) and/or sampling effects. Sparser sampling (as in our data set) may produce node density effects (see Heath *et al.* 2008; S. Y. Ho, University of Sydney, Australia, pers. comm.), but undersampling of a specific clade is not expected to affect the age of the subtending node (see Linder *et al.* 2005). In addition, the lack of other members of *Triodia* (such as a potentially faster evolving northern group) in our data set might have biased the age of the crown to be younger (see Beaulieu *et al.* 2015). Regardless, using either their or our date at least rules out rapid radiation of the complex since the earliest evidence of sandy dunefields *c*. 1 Ma (Fujioka *et al.* 2009).

### Synthesis: historical biogeography of the *T. basedowii* complex

Since the ancestors of *Triodia* arrived in Australia, probably around the mid-Miocene and in the south-west of the continent, major clades have diverged as the genus spread northwards (Toon *et al.* 2015). The clade to which the *T. basedowii* complex belongs diverged prior to the bulk of diversification in *Triodia* and currently occupies areas in central Australia, between and overlapping the distributions of (older) southern and (more recently diverged) northern clades. The two closest relatives of the *T. basedowii* complex (*T. plurinervata* and *T. concinna*) are currently distributed on the west coast and in the central interior of Western Australia (Anderson *et al.* 2017a), suggesting that ancestors of the *T. basedowii* complex likely occurred in central and western Western Australia.

**Figure 4.**
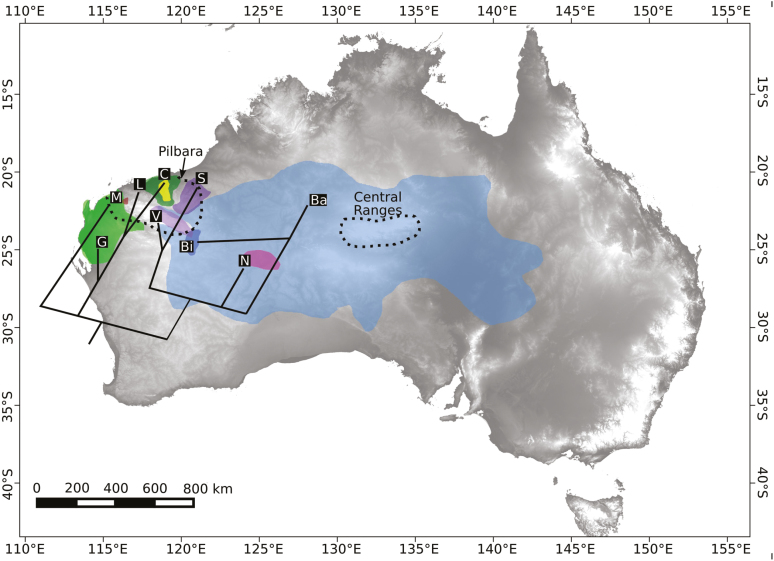
Current distributions of taxa in the *Triodia basedowii* complex, with phylogenetic relationships superimposed. Two putative refugia, the Pilbara and the Central Ranges, are labelled and denoted with dotted lines. Taxa are denoted by letters, where G: *T. glabra*, M: *T. mallota*, L: *T. lanigera*, C: *T*. *chichesterensis*, V: *T. vanleeuwenii*, S: *T. scintillans*, Bi: *T. birriliburu*, N: *T. nana* and Ba: *T. basedowii*.

While phylogenetic relationships in the complex remain partly unresolved, analyses of genomic data (Anderson *et al.* 2017b) indicate two main groups in the complex (Fig. 4): a western group (*T. mallota*, *T. glabra*, *T. lanigera* and *T*. *chichesterensis*) and an eastern group (*T. basedowii*, *T. birriliburu*, *T. vanleeuwenii*, *T. scintillans* and *T. nana*). Some of these relationships are supported by the chloroplast data presented in this study, although *T. vanleeuwenii* samples have been found with both of the two main chloroplast haplotypes, possibly due to chloroplast capture or incomplete lineage sorting (Anderson *et al.* 2016). We speculate that early divergences in the complex included a split between predominantly western and eastern lineages.

Substrate and potentially ecological differentiation have probably influenced divergences in the complex (Anderson *et al.* 2016). Western lineages may have diversified along the west coast of the continent and into the northern Pilbara (see Fig. 4), regions that currently have different substrates (sandy coastal plains vs. gravelly plains). Current habitat preferences of allopatric *T. glabra* (sandy) compared to *T*. *chichesterensis* (gravelly) suggest ecological differentiation may have developed over time in isolation, while the contrasting sympatric pattern of *T. lanigera* and *T*. *chichesterensis* (less pronounced substrate preferences) might be explained by divergence in isolation. Eastern lineages, too, show strong substrate preference differences, with three species that are largely restricted to rocky substrates (*T. vanleeuwenii*, *T. scintillans* and *T. nana*) and two that are found on sandy substrates (*T. basedowii* and *T. birriliburu*). The extent of sandy habitats prior to the formation of the dunefields *c*. 1 Ma is currently unknown, and it is possible that the ancestors of the sand specialists had available habitat in central Australia. We speculate that the eastern lineage split into a sandy lineage and a rocky lineage, the latter occupying rocky habitats and entering the south-east Pilbara.

The timing of the restriction of *T. basedowii* to central or eastern Australia is unclear, though climatic changes around the formation of the sandy dunefields *c*. 1 Ma could be reasonably implicated. Whether the split of *T. basedowii* from its sister *T. birriliburu* was coincident with that isolation or occurred after a later expansion from the east is also not clear. The westward expansion of *T. basedowii* across new sandy habitat may reflect an initial colonization of the dunefields followed by persistence through glacial cycles, or possibly a series of range expansions, with regional genetic differentiation from range restrictions or isolation by distance.

Despite the lack of precision around the timing of evolutionary events in the *T. basedowii* complex, distribution patterns illustrate that lineages have responded variably to the effects of aridity and associated landscape changes. Even among these close relatives, some are restricted to narrow ranges associated with specific rocky substrates, while others have adapted to expand onto newer sandy dunefield habitats, ultimately occupying vast areas of inland Australia. Future discoveries around the extent and types of habitats and substrates available to plants in the AAZ, and the amount of vegetation cover, from the onset of aridity in the mid-Miocene through Plio-Pleistocene climate changes will improve our understanding of adaptation, colonization and evolution within the AAZ.

## Supporting Information

Supporting information may be found in the online version of this article, and includes the following: (i) Sampling (**Tables S1** and **S2**), range expansion results (Table S3) and additional details for divergence dating analyses (including **Tables S4**–**S6** and **Figs S1**–**S5**); (ii) chloroplast alignments for data sets 1 and 2; and (iii) custom scripts.

Demultiplexed genotyping-by-sequencing reads are available on the NCBI Sequence Read Archive (SRA) under BioProject PRJNA350598, samples SAMN05942208–SAMN05942351. GenBank accession numbers for newly sequenced chloroplasts are included in **Table S2**.

Supplementary Data1

Supplementary Data2

Supplementary Materials

## Sources of Funding

This work was supported by the Australian Research Council (Linkage Project LP120100350 to CIs Grierson, Krauss *et al*. in collaboration with Rio Tinto Iron Ore, Chevron Australia Pty Ltd, Botanic Gardens and Parks Authority and the Department of Parks and Wildlife to P.F.G., S.L.K., Charles Price, Kingsley Dixon, and K.R.T.); an ANZ Trustees Foundation—Holsworth Wildlife Research Endowment to B.M.A.; and the University of Western Australia (International Postgraduate Research Scholarship, Australian Postgraduate Award and a UWA Top-up scholarship to B.M.A.).

## Conflict of Interest

None declared.

## Contributions by the Authors

B.M.A. and M.D.B. conceived the study; B.M.A., M.D.B., K.R.T., and P.G.N. collected genetic material; P.G.N. and M.D.B. sequenced chloroplast genomes; I.D.S., X.Z., and M.D.B. assembled chloroplast genomes; I.D.S., X.Z., and M.D.B. annotated chloroplast genomes; B.M.A. wrote the scripts and analysed the data; P.F.G., S.L.K., K.R.T., M.D.B., and B.M.A. interpreted results; B.M.A. led the writing.
