# Supplementary material for: Recent range expansion in Australian hummock grasses (Triodia) inferred using genotyping-by-sequencing
Source: AoB Plants. 2019 Mar 28;11(2):plz017. doi: 10.1093/aobpla/plz017 (PMC6481909; doi:10.1093/aobpla/plz017)
Supplement: Supplementary Materials [file plz017_suppl_supplementary_materials.pdf]

Supporting Information for "Recent range expansion in Australian hummock grasses (*Triodia*) inferred using genotyping-by-sequencing"

BM Anderson, KR Thiele, PF Grierson, SL Krauss, PG Nevill, ID Small, X Zhong and MD Barrett

Table of Contents

Sampling....2
Table S1. Samples used in the range expansion analyses....2
Table S2. Chloroplast genomic samples used in divergence dating analyses....4
Range expansion results....6
Table S3....6
Additional details for divergence dating analyses....9
MATERIALS AND METHODS....9
Chloroplast genome sequencing and assembly....9
Phylogenetic analysis....11
Divergence dating....12
RESULTS....14
Table S4....17
Table S5....18
Table S6....18
Figure S1....19
Figure S2....20
Figure S3....21
Figure S4....22
Figure S5....23
SUPPORTING INFORMATION LITERATURE CITED....24

Sampling

Table S1. Samples used in the range expansion analyses.

Sample ID	Species	Voucher Collector	Coll. #	Locality	Latitude	Longitude	Accession #
Base-bas-Andado05	Triodia basedowii	B.M. Anderson & P. Jobson	BMA 80	Old Andado Hstd, NT	25° 16' 29" S	135° 24' 37" E	SAMN05942269
Base-bas-Andado07	Triodia basedowii	B.M. Anderson & P. Jobson	BMA 80	Old Andado Hstd, NT	25° 16' 29" S	135° 24' 37" E	SAMN05942270
Base-bas-Andado12	Triodia basedowii	B.M. Anderson & P. Jobson	BMA 80	Old Andado Hstd, NT	25° 16' 29" S	135° 24' 37" E	SAMN05942272
Base-bas-Andado18	Triodia basedowii	B.M. Anderson & P. Jobson	BMA 80	Old Andado Hstd, NT	25° 16' 29" S	135° 24' 37" E	SAMN05942273
Base-bas-BMA53	Triodia basedowii	B.M. Anderson & K. Thiele	BMA 53	Gunbarrel Hwy, WA	25° 42' 15.4" S	125° 42' 47.6" E	SAMN05942274
Base-bas-BMA77	Triodia basedowii	B.M. Anderson & P. Jobson	BMA 77	Maryvale Stn, NT	24° 42' 16" S	134° 6' 7" E	SAMN05942275
Base-bas-Erl02	Triodia basedowii	B.M. Anderson & P. Jobson	BMA 81	Erldunda Jnctn, NT	25° 11' 59" S	133° 12' 0" E	SAMN05942276
Base-bas-Ghan14	Triodia basedowii	B.M. Anderson & P. Jobson	BMA 79	Finke, NT	25° 7' 37" S	134° 23' 40" E	SAMN05942277
Base-bas-Huck03	Triodia basedowii	B.M. Anderson, M.D. Barrett & P. Jobson	BMA 72	Huckitta Hstd, NT	22° 54' 22" S	135° 26' 43" E	SAMN05942278
Base-bas-Lara06	Triodia basedowii	B.M. Anderson, M.D. Barrett & P. Jobson	BMA 75	Larapinta Rd, NT	23° 52' 58" S	132° 26' 43" E	SAMN05942279
Base-bas-Lara13	Triodia basedowii	B.M. Anderson, M.D. Barrett & P. Jobson	BMA 75	Larapinta Rd, NT	23° 52' 58" S	132° 26' 43" E	SAMN05942280
Base-bas-Lara19	Triodia basedowii	B.M. Anderson, M.D. Barrett & P. Jobson	BMA 75	Larapinta Rd, NT	23° 52' 58" S	132° 26' 43" E	SAMN05942281
Base-bas-Nar09	Triodia basedowii	B.M. Anderson, M.D. Barrett & P. Jobson	BMA 74	Narwietooma Stn, NT	23° 10' 53" S	132° 39' 57" E	SAMN05942282
Base-bas-Niftyb06	Triodia basedowii	B.M. Anderson	BMA 37	Nifty copper mine, WA	21° 39' 49.7" S	121° 33' 41.5" E	SAMN05942216
Base-bas-Niftyb13	Triodia basedowii	B.M. Anderson	BMA 37	Nifty copper mine, WA	21° 39' 49.7" S	121° 33' 41.5" E	SAMN05942217
Base-bas-Niftyb18	Triodia basedowii	B.M. Anderson	BMA 37	Nifty copper mine, WA	21° 39' 49.7" S	121° 33' 41.5" E	SAMN05942283
Base-bas-NNew07	Triodia basedowii	B.M. Anderson & M.D. Barrett	BMA 31	Roy Hill, WA	22° 47' 16.1" S	120° 0' 14.3" E	SAMN05942284
Base-bas-Olga13	Triodia basedowii	B.M. Anderson & P. Jobson	BMA 83	W of the Olgas, NT	25° 10' 0" S	130° 25' 8" E	SAMN05942285
Base-bas-Roy1b03	Triodia basedowii	M.D. Barrett & W. Lewandrowski	MDB 3932	E of Munjina, WA	22° 26' 35" S	118° 54' 31.1" E	SAMN05942286
Base-bas-Roy1b08	Triodia basedowii	M.D. Barrett & W. Lewandrowski	MDB 3932	E of Munjina, WA	22° 26' 35" S	118° 54' 31.1" E	SAMN05942288
Base-bas-Roy1b12	Triodia basedowii	M.D. Barrett & W. Lewandrowski	MDB 3932	E of Munjina, WA	22° 26' 35" S	118° 54' 31.1" E	SAMN05942289
Base-bas-Roy1b15	Triodia basedowii	M.D. Barrett & W. Lewandrowski	MDB 3932	E of Munjina, WA	22° 26' 35" S	118° 54' 31.1" E	SAMN05942290
Base-bas-Roy1b20	Triodia basedowii	M.D. Barrett & W. Lewandrowski	MDB 3932	E of Munjina, WA	22° 26' 35" S	118° 54' 31.1" E	SAMN05942291
Base-bas-Stirling12	Triodia basedowii	M.D. Barrett, B.M. Anderson & P. Jobson	MDB 4520	Stirling Hstd, NT	21° 39' 38" S	133° 44' 47" E	SAMN05942292
Base-basswool-BMA41	Triodia basedowii	B.M. Anderson & K. Thiele	BMA 41	E of Wiluna, WA	26° 39' 11.5" S	121° 6' 57.7" E	SAMN05942299
Base-basswool-Katb03	Triodia basedowii	B.M. Anderson, S. van Leeuwen, N. Gibson, M. Langley & K. Brown	BMA 66	Carnarvon Range, WA	25° 2' 59" S	120° 44' 5.3" E	SAMN05942300
Base-basswool-Katb07	Triodia basedowii	B.M. Anderson, S. van Leeuwen, N. Gibson, M. Langley & K. Brown	BMA 66	Carnarvon Range, WA	25° 2' 59" S	120° 44' 5.3" E	SAMN05942301
Base-basswool-Katb13	Triodia basedowii	B.M. Anderson, S. van Leeuwen, N. Gibson, M.	BMA 66	Carnarvon Range, WA	25° 2' 59" S	120° 44' 5.3" E	SAMN05942302

Sample ID	Species	Voucher Collector	Coll. #	Locality	Latitude	Longitude	Accession #
		Langley & K. Brown					
Base-basswool-Katb19	Triodia basedowii	B.M. Anderson, S. van Leeuwen, N. Gibson, M. Langley & K. Brown	BMA 66	Carnarvon Range, WA	25° 2' 59" S	120° 44' 5.3" E	SAMN05942303
Base-basswool-Leinster07	Triodia basedowii	B.M. Anderson, R. Davis & T. Hammer	BMA 4	Leinster, WA	28° 5' 16.6" S	120° 53' 34" E	SAMN05942304
Base-bas-TB1-02	Triodia basedowii	T.E. Erickson	TEE 771	Capricorn Roadhouse, WA	23° 33' 13.9" S	119° 45' 35.1" E	SAMN05942293
Base-bas-TB1-09	Triodia basedowii	T.E. Erickson	TEE 771	Capricorn Roadhouse, WA	23° 33' 13.9" S	119° 45' 35.1" E	SAMN05942294
Base-bas-TB1-13	Triodia basedowii	T.E. Erickson	TEE 771	Capricorn Roadhouse, WA	23° 33' 13.9" S	119° 45' 35.1" E	SAMN05942295
Base-bas-TB1-17	Triodia basedowii	T.E. Erickson	TEE 771	Capricorn Roadhouse, WA	23° 30' 2.1" S	119° 46' 56.5" E	SAMN05942296
Base-bas-Tjuk14	Triodia basedowii	B.M. Anderson & K. Thiele	BMA 58	S of Tjukayirla, WA	27° 49' 28.3" S	123° 56' 50.1" E	SAMN05942297
Base-bas-Tjuk17	Triodia basedowii	B.M. Anderson & K. Thiele	BMA 58	S of Tjukayirla, WA	27° 49' 28.3" S	123° 56' 50.1" E	SAMN05942298
Base-lsandy-Kat10	Triodia birriliburu	B.M. Anderson, S. van Leeuwen, N. Gibson, M. Langley & K. Brown	BMA 62	Carnarvon Range, WA	25° 2' 47.8" S	120° 44' 25.7" E	SAMN05942315
Base-lsandy-Kat15	Triodia birriliburu	B.M. Anderson, S. van Leeuwen, N. Gibson, M. Langley & K. Brown	BMA 62	Carnarvon Range, WA	25° 2' 47.8" S	120° 44' 25.7" E	SAMN05942316
Base-lsandy-Kat16	Triodia birriliburu	B.M. Anderson, S. van Leeuwen, N. Gibson, M. Langley & K. Brown	BMA 62	Carnarvon Range, WA	25° 2' 47.8" S	120° 44' 25.7" E	SAMN05942317
Base-nana-Gibson02	Triodia nana	B.M. Anderson & K. Thiele	BMA 49	Gunbarrel Hwy, WA	25° 21' 13.7" S	124° 26' 46.2" E	SAMN05942318
Base-nana-Gibson05	Triodia nana	B.M. Anderson & K. Thiele	BMA 49	Gunbarrel Hwy, WA	25° 21' 13.7" S	124° 26' 46.2" E	SAMN05942319
Base-nana-Gibson15	Triodia nana	B.M. Anderson & K. Thiele	BMA 49	Gunbarrel Hwy, WA	25° 21' 13.7" S	124° 26' 46.2" E	SAMN05942320
Base-nana-Gibson19	Triodia nana	B.M. Anderson & K. Thiele	BMA 49	Gunbarrel Hwy, WA	25° 21' 13.7" S	124° 26' 46.2" E	SAMN05942321
Base-peed-Stew11	Triodia glabra	M.D. Barrett & W. Lewandrowski	MDB 3978	W of Paraburdoo, WA	22° 25' 47.6" S	115° 56' 35.5" E	SAMN05942242
Base-peed-Stew13	Triodia glabra	M.D. Barrett & W. Lewandrowski	MDB 3978	W of Paraburdoo, WA	22° 25' 47.6" S	115° 56' 35.5" E	SAMN05942243
Base-peed-Uaroo01	Triodia glabra	M.D. Barrett & B.M. Anderson	MDB 4120	SW of Nanutarra, WA	22° 46' 11" S	115° 6' 37" E	SAMN05942322
Base-peed-Uaroo03	Triodia glabra	M.D. Barrett & B.M. Anderson	MDB 4120	SW of Nanutarra, WA	22° 46' 11" S	115° 6' 37" E	SAMN05942323
Base-peed-Uaroo05	Triodia glabra	M.D. Barrett & B.M. Anderson	MDB 4120	SW of Nanutarra, WA	22° 46' 11" S	115° 6' 37" E	SAMN05942324

Table S2. Chloroplast genomic samples used in divergence dating analyses.

Subfamily	Species	Source	Data set 1	Data set 2	Genbank accession	Herb.	Barcode	Voucher Collector	Coll. #	Locality	Latitude	Longitude
Anomochloideae	Anomochloa marantoidea	Download	yes		NC_014062							
Aristidoideae	Sartidia perrieri	Download	yes		KJ819549							
Arundinoideae	Monachather paradoxus	Download	yes		KJ920235							
Arundinoideae	Phragmites australis	Download	yes		KJ825856							
Bambusoideae	Arundinaria appalachiana	Download	yes		KC817462							
Bambusoideae	Bambusa oldhamii	Download	yes		NC_012927							
Bambusoideae	Olyra latifolia	Download	yes		KF515509							
Chloridoideae	Neyraudia reynaudiana	Download	yes	yes	KF356392							
Chloridoideae	Sporobolus maritimus	Download	yes	yes	KP176438							
Chloridoideae	Zoysia macrantha	Download		yes	KT168390							
Danthonioideae	Chionochloa macra	Download	yes	yes	KJ920227							
Danthonioideae	Danthonia californica	Download	yes	yes	KJ920229							
Micrairoideae	Isachne distichophylla	Download	yes		KJ920233							
Micrairoideae	Micraira sp. JLC-2014	Download	yes		KJ920234							
Oryzoideae	Chikusichloa aquatica	Download	yes		NC_027184							
Oryzoideae	Microlaena stipoides	Download	yes		GU592211							
Oryzoideae	Oryza sativa Japonica Group	Download	yes		NC_001320							
Oryzoideae	Zizania latifolia	Download	yes		KT161956							
Panicoideae	Centotheca lappacea	Download	yes		KJ920225							
Panicoideae	Setaria viridis	Download	yes		KT289405							
Panicoideae	Sorghum bicolor	Download	yes		NC_008602							
Pharoideae	Pharus latifolius	Download	yes		JN032131							
Pooideae	Brachyelytrum aristosum	Download	yes		KM974735							
Pooideae	Poa palustris	Download	yes		KM974749							
Pooideae	Stipa hymenoides	Download	yes		KM974729							
Puelioideae	Puelia olyriformis	Download	yes		KC534841							
Aristidoideae	Aristida pruinosa	New	yes		MK590083	PERTH	8446334	S. Dillon & A. Markey	CR 9260	Cane River Conservation Park, WA	21° 59' 59.3" S	115° 51' 40.3" E
Micrairoideae	Eriachne tenuiculmis	New	yes		MK590078	PERTH	8162107	B. Cook	C 27-04	Coondiner Mining tenement, WA	22° 58' 22" S	119° 36' 30" E
Chloridoideae	Aeluropus lagopoides	New		yes	MK622397	E	E00358268	J. Martin	176	UAE		
Chloridoideae	Astrebla lappacea	New	yes	yes	MK590086	PERTH	8353697	M.S. Trudgen & P. Hoffmann	BES PH 116	NW of Tom Price, WA	22° 17' 10.6" S	117° 14' 39.9" E
Chloridoideae	Cleistogenes sp.	New		yes	MK622398	E	E00639685	V. Goloskokov	s.n.	Kazakhstan		
Chloridoideae	Dactyloctenium radulans	New	yes	yes	MK590085	PERTH	8377332	B. Vincent & N. Krawczyk	BV 141	WNW of Paraburdoo, WA	22° 54' 50.6" S	117° 7' 9.7" E

Subfamily	Species	Source	Data set 1	Data set 2	Genbank accession	Herb.	Barcode	Voucher Collector	Coll. #	Locality	Latitude	Longitude
Chloridoideae	Enneapogon caerulescens	New	yes	yes	MK590084	PERTH	8127085	V. Long	VL 1494 39	E of Burrup Road, WA	20° 38' 18.3" S	116° 46' 9" E
Chloridoideae	Enteropogon ramosus	New	yes	yes	MK590081	PERTH	8446393	S. Dillon & A. Markey	CR 9267	Cane River Conservation Park, WA	22° 13' 11.7" S	115° 27' 19.2" E
Chloridoideae	Eragrostis setifolia	New		yes	MK590079	PERTH	6437788	S. van Leeuwen	4683	Coondewanna Flats, WA	23° 1' 15.2" S	118° 45' 24.9" E
Chloridoideae	Eragrostis tenellula	New	yes	yes	MK590080	PERTH	8377391	J. Atkinson	JA 95	WNW of Paraburdoo, WA	22° 25' 28.2" S	116° 18' 41.5" E
Chloridoideae	Orinus kokonorica	New		yes	MK622399	E	E00064287	Sino-American-British Yushu Expedition (1996)	2759	China	32° 17' N	96° 28' E
Chloridoideae	Tragus australianus	New	yes	yes	MK590077	PERTH	8446253	S. Dillon & A. Markey	CR 9282	Cane River Conservation Park, WA	22° 10' 11.8" S	115° 33' 39.6" E
Chloridoideae	Triodia basedowii	New	yes	yes	MK622400	PERTH	8618860	M.D. Barrett & B.M. Anderson	MDB 4127	Munjina, WA	22° 20' 17.8" S	118° 35' 46.5" E
Chloridoideae	Triodia chichesterensis	New		yes	MK622401	PERTH	8618852	M.D. Barrett & B.M. Anderson	MDB 4102	Boodarie, WA	20° 41' 15" S	118° 41' 11.8" E
Chloridoideae	Triodia concinna	New		yes	MK622402	PERTH	-	B.M. Anderson & K. Thiele	BMA 45	E of Carnegie Hstd, WA	25° 25' 38.4" S	123° 46' 20.1" E
Chloridoideae	Triodia glabra	New		yes	MK622403	PERTH	8618968	M.D. Barrett & W. Lewandrowski	MDB 3978	Mt Stuart, WA	22° 25' 48.1" S	115° 56' 19.1" E
Chloridoideae	Triodia lanigera	New	yes	yes	MK622413	PERTH	8618895	M.D. Barrett & B.M. Anderson	MDB 4099	Marble Bar, WA	20° 39' 54.1" S	119° 13' 13" E
Chloridoideae	Triodia longiceps	New	yes	yes	MK622404	PERTH	8618925	M.D. Barrett & B.M. Anderson	MDB 4103	Boodarie, WA	20° 44' 24.9" S	118° 40' 57.2" E
Chloridoideae	Triodia mallota	New		yes	MK622405	PERTH	8618909	B.M. Anderson & M.D. Barrett	BMA 89	Pannawonica, WA	21° 45' S	116° 18' E
Chloridoideae	Triodia plurinervata	New		yes	MK622406	PERTH	-	C.E. Mayence	s.n.	Shark Bay, WA	26° 10' 14" S	113° 23' 25.2" E
Chloridoideae	Triodia rigidissima	New		yes	MK622407	PERTH	8957649	M.D. Barrett & B.M. Anderson	MDB 3994	Yellowdine, WA	31° 15' 28.5" S	120° 2' 25.7" E
Chloridoideae	Triodia schinzii	New	yes	yes	MK622410	PERTH	8618992	M.D. Barrett & B.M. Anderson	MDB 4375	Broome, WA	17° 58' 30.4" S	122° 10' 50.3" E
Chloridoideae	Triodia scintillans	New		yes	MK622411	PERTH	8618887	M.D. Barrett & W. Lewandrowski	MDB 3944	Kulbee Ck, WA	22° 23' 13.5" S	119° 58' 31.5" E
Chloridoideae	Triodia tomentosa	New		yes	MK622408	PERTH	-	M.D. Barrett & B.M. Anderson	MDB 3990	Yellowdine, WA	31° 17' 35.1" S	119° 39' 6.9" E
Chloridoideae	Triodia vanleeuwenii	New		yes	MK622409	PERTH	8618879	M.D. Barrett & W. Lewandrowski	MDB 3969	Karijini, WA	22° 34' 28.5" S	118° 12' 54.1" E
Chloridoideae	Triodia wiseana	New		yes	MK622412	PERTH	8618763	M.D. Barrett & B.M. Anderson	MDB 4086	Telfer Mine Road, WA	21° 18' 10" S	121° 12' 1" E
Chloridoideae	Tripogon loliiformis	New	yes	yes	MK590087	PERTH	8415153	K.R. Thiele	3969	Pianto Road, WA	29° 54' 6" S	121° 16' 37" E
Chloridoideae	Triraphis mollis	New	yes	yes	MK590082	PERTH	7851472	R. Orifici	89	SSW of Mt Brockman, WA	22° 33' 46.5" S	117° 14' 24.7" E

Range expansion results

Table S3.

Results from running the range expansion analyses for variations in filtering SNPs and samples. SNP states were polarized using different outgroups: *Triodia birriliburu*, *T. nana*, and *T. glabra.* Loci sets were (1) assembled, (2) unassembled, or (3) a combination of the two. SNPs were chosen (i) randomly or (ii) with a bias toward SNPs with multiple copies of the rare allele. Sampling subsets are I: one per population (random), II: one per population (alternative choice), III: one per population excluding tetraploids, IV: all samples (populations downsampled), and V: all samples excluding tetraploids (populations downsampled). The number of polarized SNPs indicates how many remain after the scripts remove those that have an ambiguous ancestral state. Geographic sample subsets are "all", "west", "eastern" and "intermediate", and the signal for expansion was deemed significant if $P < 0.01$. NT is the Northern Territory, SA is South Australia, and QLD is Queensland.

Outgroup	SNP set	SNP choice	Sampling	Samples	polarized SNPs	Sig Reg	P-value	Location	Eastern origin?
T. birriliburu	1	i	I	18	2353	all	6.53E-09	SE NT	Y
			II	18	2395	-			
			III	15	2101	all	7.64E-12	S NT	Y
			IV	36	3409	all	7.99E-17	S NT	Y
			V	27	2913	all	1.18E-19	NE SA	Y
		ii	I	18	2397	all	8.74E-03	N NT	Y
			II	18	2393	all	1.06E-06	S NT	Y
			III	15	2136	-			
			IV	36	3387	all	7.04E-17	S NT	Y
			V	27	2872	all	7.83E-19	SE NT	Y
	2	i	I	18	2902	all	1.14E-05	NW QLD	Y
			II	18	2958	-			
			III	15	2570	all	1.59E-06	NE NT	Y
			IV	36	4193	all	1.16E-11	NE SA	Y
			V	27	3460	all	7.87E-10	W QLD	Y
		ii	I	18	2978	all	1.08E-04	NW QLD	Y
			II	18	2991	all	8.07E-07	NW QLD	Y
			III	15	2631	all	5.02E-05	NW QLD	Y
			IV	36	4201	all	7.89E-14	E NT	Y
			V	27	3515	all	2.30E-13	W QLD	Y
	3	i	I	18	5255	all	3.58E-08	W QLD	Y
			II	18	5353	all	9.57E-03	NW QLD	Y
			III	15	4671	all	3.92E-11	SE NT	Y

Outgroup	SNP set	SNP choice	Sampling	Samples	polarized SNPs	Sig Reg	P-value	Location	Eastern origin?
			IV	36	7602	all	4.06E-15	S NT	Y
			V	27	6373	all	9.91E-16	NE SA	Y
		ii	I	18	5375	all	4.35E-06	NE NT	Y
			II	18	5384	all	1.01E-08	NW QLD	Y
			III	15	4767	all	3.04E-06	NW QLD	Y
			IV	36	7588	all	3.62E-17	S NT	Y
			V	27	6387	all	4.75E-18	W QLD	Y
T. nana	1	i	I	18	2326	all	2.50E-10	W QLD	Y
			II	18	2332	all	2.75E-09	NW QLD	Y
			III	15	2075	all	1.89E-12	NW QLD	Y
			IV	36	3251	all	1.97E-19	S NT	Y
			V	27	2737	all	3.13E-20	W QLD	Y
	1	ii	I	18	2323	-			
			II	18	2337	all	2.15E-07	S NT	Y
			III	15	2055	all	1.15E-05	E NT	Y
			IV	36	3282	all	1.64E-13	E NT	Y
			V	27	2758	all	7.46E-20	W QLD	Y
	2	i	I	18	2830	-			
			II	18	2848	all	2.27E-03	NE NT	Y
			III	15	2494	-			
			IV	36	3917	all	6.89E-12	NW QLD	Y
			V	27	3229	all	1.59E-12	NW QLD	Y
	2	ii	I	18	2761	**eastern**	1.97E-04	S NT	**N**
			II	18	2802	-			
			III	15	2396	-			
			IV	36	3876	all	1.55E-16	E NT	Y
			V	27	3198	all	3.04E-21	W QLD	Y
	3	i	I	18	5156	all	1.64E-08	NW QLD	Y
			II	18	5180	all	2.07E-09	NW QLD	Y
			III	15	4569	all	3.25E-11	NE NT	Y
			IV	36	7168	all	5.30E-18	E NT	Y
			V	27	5966	all	8.63E-20	W QLD	Y
	3	ii	I	18	5084	-			
			II	18	5139	all	4.14E-08	NW QLD	Y
			III	15	4451	all	2.81E-05	NW QLD	Y
			IV	36	7158	all	2.16E-16	E NT	Y
			V	27	5956	all	2.62E-24	W QLD	Y

Outgroup	SNP set	SNP choice	Sampling	Samples	polarized SNPs	Sig Reg	P-value	Location	Eastern origin?
T. glabra	1	i	I	18	2099	all	2.27E-03	S NT	Y
			II	18	2145	all	9.58E-04	S NT	Y
			III	15	1883	all	8.38E-04	S NT	Y
			IV	36	2973	all	3.72E-11	S NT	Y
			V	27	2553	all	1.83E-11	S NT	Y
	1	ii	I	18	2144	-			
			II	18	2170	-			
			III	15	1928	-			
			IV	36	2966	all	3.59E-13	S NT	Y
			V	27	2553	all	6.50E-13	SE NT	Y
	2	i	I	18	2727	-			
			II	18	2686	-			
			III	15	2424	all	9.38E-07	central NT	Y
			IV	36	3714	all	4.94E-09	E NT	Y
			V	27	3136	all	8.64E-12	NW QLD	Y
	2	ii	I	18	2759	-			
			II	18	2767	-			
			III	15	2464	all	1.32E-03	S NT	Y
			IV	36	3706	all	1.48E-06	S NT	Y
			V	27	3149	all	9.37E-09	W QLD	Y
	3	i	I	18	4826	all	5.31E-04	NW NT	Y
			II	18	4831	all	1.27E-02	NW QLD	Y
			III	15	4307	all	2.60E-08	central NT	Y
			IV	36	6687	all	5.70E-12	S NT	Y
			V	27	5689	all	3.54E-15	W QLD	Y
	3	ii	I	18	4903	-			
			II	18	4937	-			
			III	15	4392	all	4.15E-04	S NT	Y
			IV	36	6672	all	1.53E-11	S NT	Y
			V	27	5702	all	1.15E-13	NE SA	Y

Additional details for divergence dating analyses

MATERIALS AND METHODS

Chloroplast genome sequencing and assembly

For divergence time estimation across Poaceae, we obtained chloroplast genomes for members of all subfamilies, 26 downloaded from GenBank and 13 newly sequenced and assembled (Table S2). We aimed to include two or three genomes from each subfamily, four for Oryzoideae and 14 for Chloridoideae, but were only able to obtain one representative from each of Anomochloideae, Pharoideae and Puelioideae. Sample classification and subfamilial taxonomy follows Soreng *et al*. (2015). For a second analysis with denser sampling of Chloridoideae and *Triodia*, we included an additional ten *Triodia* and five other Chloridoideae genomes newly sequenced and assembled.

Silica-dried leaf material was ground in liquid nitrogen and genomic DNA extracted using a CTAB method (Doyle and Dickson 1987) as described in Anderson *et al*. (2016) but with RNAse added prior to heating. DNA was also extracted from herbarium samples using the commercial DNeasy Plant Mini Kit (Qiagen, USA) following manufacturer's instructions, with DNA eluted in 100 µl of AE buffer. DNA concentration was quantified on a NanoDrop ND-1000 spectrophotometer and samples with concentrations of 3–20 ng/µl sent to the Australian Genome Research Facility node in Melbourne, Victoria. 200 ng of DNA was sheared in a volume of 50 µl using a Coavris E220 Focused ultrasonicator. Following shearing, sequencing libraries were prepared using Illumina's TruSeq Nano DNA Library preparation kit (350 bp median insert) following the manufacturer's protocol. Libraries were assessed by gel electrophoresis (Agilent D1000 ScreenTape Assay) and quantified by qPCR (KAPA Library Quantification Kits for Illumina). Sequencing was performed on the Illumina

HiSeq 2500 system with 2×125 bp paired-end reads using the HiSeq PE Cluster Kit v. 5 and HiSeq SBS Kit v. 4 (250 cycles).

For 19 genomes, adapter sequences ("AGATCGGAAGAGCACACGTCTGAACTCCAGTCAC" and "AGATCGGAAGAGCGTCGTGTAGGGAAAGAGTGTAGATCTCGGTGGTCGCCGTATCATT") in raw paired-end reads were removed using the software CUTADAPT v. 1.9.1 (Martin 2011). Sequencing read errors were corrected with SPAdes v. 3.6.1 (Bankevich *et al.* 2012). The filtered reads were normalised for sequencing depth based on k-mer counts and merged into single reads using BBNorm and BBMerge, respectively, from the BBMap package v. 35.82 (https://sourceforge.net/projects/bbmap/ ; visited June 2016). The processed reads were assembled into contigs using Velvet v. 1.2.09 (Zerbino and Birney 2008) with k-mer values 51, 71, 91 and 111, and coverage cut-off values 7, 10, 15 and 20. Assembled contigs were aligned to the chloroplast genome of *Spinacia oleracea* (GenBank accession NC_002202) using MUMmer v. 3.1 (Kurtz *et al.* 2004). Based on the alignments, assembled chloroplast contigs were identified, ordered and then merged into a single circular draft genome for each specimen using a custom script. Reads were mapped back to the assembly using BWA v. 0.7.5a-r405 (Li and Durbin 2009) and the assembly further refined with Pilon v. 1.16 (Walker *et al.* 2014). The *Setaria viridis* genome (GenBank accession NC_028075) was used as a reference for gene annotation.

One chloroplast genome (*T. vanleeuwenii*) was sequenced using 454 shotgun sequencing by M. Gardner, using the method outlined in Gardner *et al*. (2011), and assembled by M. Duvall, S. Burke and W. Wysocki, (Northern Illinois University) using plastome assembly and verification methods outlined in Wysocki *et al*. (2014) and Duvall *et al*. (2016).

The remaining eight genomes were assembled using a beta-test version of the de novo assembler NOVOPlasty (available at: https://github.com/ndierckx/NOVOPlasty, verified

August 2017) (Dierckxsens *et al.* 2017), using various conserved seed sequences where necessary. Genomes were validated by mapping raw reads onto the assembly in Geneious v. 6.1.8 (http://www.geneious.com, Kearse *et al.* 2012) to check for ambiguously aligned regions. The NOVOPlasty algorithm was applied to three of the genomes assembled using the first method above, to demonstrate its utility in *Triodia*. The three genomes assembled using the two methods differed in, at most, a few heterozygous/homozygous calls and/or the number of units in repeat regions.

Phylogenetic analysis

We created two datasets for phylogenetic and divergence dating analyses: (1) "all grasses" (39 samples from across Poaceae with focused sampling in Chloridoideae), and (2) "chloridoids" (29 samples of Chloridoideae with focused sampling in *Triodia* plus two samples of Danthonioideae as an outgroup). Dataset 1 comprised only chloroplast coding regions, while dataset 2 included coding regions as well as introns and intergenic spacers. Whole chloroplast genomes were aligned in Geneious v. 6.1.8 (http://www.geneious.com, Kearse *et al.* 2012) using the MAFFT (Katoh and Standley 2013) plugin with gap creation 2.0 and gap extension 0.12. Target regions were manually extracted from the alignment and checked by eye for alignment ambiguities, which were removed or adjusted. A custom Python v. 2.7.2 (Python Software Foundation 2016) script was used to remove positions in the alignment with more than one missing or 'N' state.

To determine the optimum partitioning and model scheme, the loci for each dataset were run through PartitionFinder v. 2.0.0-pre13 (Lanfear *et al.* 2012), using models available for MrBayes, the Bayesian information criterion to select the optimal model, and a greedy search. The optimal partitioning scheme (see Table S4) was used in phylogenetic inference with RAxML v. 8.1.21 (Stamatakis 2014), which implements the GTRGAMMA model for all

partitions. RAxML was run with a 100 replicate rapid bootstrap (Stamatakis *et al.* 2008) followed by a search for the maximum likelihood tree ('-f a' option). We also ran the partitions and best models under Bayesian inference in MrBayes v. 3.2.6 (Ronquist *et al.* 2012). MrBayes was executed for three runs, each using four chains and two million generations, sampling every 500, with 10% of samples discarded as burn-in. Trees from each run were combined into a single file with a custom script, and a maximum clade credibility tree constructed using TreeAnnotator v. 2.4.0 (Bouckaert *et al.* 2014) and visualized in FigTree v. 1.4.3 (Rambaut 2016).

Divergence dating

We included five fossils and a root prior (Table S5) in Bayesian analyses of dataset 1 (all grasses) in BEAST v. 2.4.6 (Bouckaert *et al.* 2014). The placement of the Prasad *et al.* (2005, 2011) phytoliths is somewhat controversial (see Christin *et al.* 2014), but has been set at crown Oryzoideae in a recent dating analysis of grasses (Burke *et al.* 2016), consistent with recently discovered fossils of a spikelet from *c*. 100 Ma (Poinar *et al.* 2015). We report our results with the phytoliths at stem Oryzeae (common ancestor of *Oryza* and *Microlaena* in our tree), but we also ran analyses with them placed conservatively at stem BOP+PACMAD (Bambusoideae, Oryzoideae, Pooideae + Panicoideae, Aristidoideae, Chloridoideae, Micrairoideae, Arundinoideae, and Danthonioideae). In setting the prior probability distributions, maximum ages were arbitrarily chosen as 1.5 times the minimum age except for stem Chloridoideae, which was extended (making a less informative prior) to account for the evidence of C_4 grasses from the Oligocene (Urban *et al.* 2010). Internal node priors were implemented using log-normal distributions with a mean of 1, an offset to the minimum age, and standard deviations set such that 95% of the probability distribution was contained within the chosen intervals. The prior on the root of the tree was set as a uniform distribution from 0 to a hard maximum age of 125 Ma, under the assumption that lineages of Poaceae were not

present prior to the earliest eudicot fossil (Doyle 1992). *Anomochloa* was fixed as the outgroup (see Bouchenak-Khelladi *et al.* 2008).

For dataset 1, BEAST was run for 120 million generations, sampling every 10,000, using the Yule model for branching under a relaxed uncorrelated log-normal clock (UCLN; Drummond *et al.* 2006), and using the optimal partitioning scheme from PartitionFinder. BEAST had difficulty starting the analysis from a random tree because of the prior for placement of the 65 Ma phytoliths at stem Oryzeae, so a tree in Newick format was provided with appropriate branch lengths for that node to initialize the analysis. Rather than fixing models for the optimum partitions, we allowed the substitution models to be sampled as part of the analysis using the bModelTest v. 1.0.4 package (Bouckaert and Drummond 2017). Log files were examined in Tracer v. 1.6.0 (Rambaut *et al.* 2014) to check that the run had reached stationarity and that effective sample sizes (ESS) were large. A nominal 10% burn-in was used, after observing that the chain had reached stationarity by that point, and a maximum clade credibility tree was created using TreeAnnotator v. 2.4.6 (Bouckaert *et al.* 2014) and visualized in FigTree v. 1.4.3 (Rambaut 2016).

For dataset 2, we calibrated the node age for crown Chloridoideae using the 95% highest posterior density (HPD) interval from the analysis of dataset 1. The prior distribution was set (see Table S4) with a gamma distribution and an offset, with the shape and scale set iteratively to closely approximate the 95% HPD from the first analysis. A recent molecular dating analysis of *Triodia* (Toon *et al.* 2015) found that a random local clocks model (RLC; Drummond and Suchard 2010) provided a better fit to account for rate changes in *Triodia* than the UCLN clock (see also Crisp *et al.* 2014), which produced younger ages in *Triodia*. We ran BEAST with the same settings as in the analysis of dataset 1, except using an RLC clock model, again using the optimal partition scheme obtained from PartitionFinder. Examining log

files in Tracer showed some instability in the MCMC chain for some parameters, with stationarity reached around 60 million generations. To obtain higher ESS of parameters, we ran a second analysis with the same settings then combined the two runs and constructed a maximum clade credibility tree after discarding appropriate burn-ins (50% and 25%, respectively).

We ran two additional analyses to assess the impact of clock model and alignment size. First, we ran the analysis of dataset 2 again with the same settings, but this time using a UCLN clock model. Examining the log file in Tracer showed stationarity after approximately 30% burn-in, so this was used to construct a maximum clade credibility tree. Second, we assessed whether differences in the dates we obtained compared to those of Toon *et al*. (2015) might be due to the length of our alignment. We extracted the *matK* region from our alignment of dataset 2 and ran an analysis with just that locus, using the same settings as in the original analysis of dataset 2 (with an RLC clock model). Examining the run in Tracer showed wide variation in the prior and posterior (with low ESS) as well as a few anomalous samples well outside the range of the chain. We combined the results with those of a second run, after discarding burn-in and removing anomalous samples with a custom script, but still had low ESS for the posterior and prior, possibly reflecting low information content in the *matK* locus. Results from the *matK* analyses should therefore be interpreted cautiously.

RESULTS

The 28 newly-assembled grass chloroplast genomes were on average *c*. 135,000 bp (132,643–137,308 bp) in length. There were no major discrepancies in gene content or order. After filtering the aligned genomic regions, dataset 1 comprised 79 loci (coding regions) with a combined length of 62,005 bp, while dataset 2 comprised 163 loci (coding regions, introns or spacers) with a combined length of 101,510 bp. Phylogenetic inference for both datasets

resulted in highly resolved trees (Figs. S1–S4), with high (100%/1.00 posterior probability) support for all grass subfamilies except Pharoideae and Anomochloideae (single samples), and high support for *Triodia* as a genus and for the *T. basedowii* species complex. Phylogenetic relationships of grass subfamilies are in agreement with the current understanding of evolution in Poaceae (Grass Phylogeny Working Group II 2012; Soreng *et al.* 2015). Topologies are identical between RAxML and MrBayes trees, aside from the lack of resolution at the base of the trees for dataset 1.

Divergence dates for important nodes across the grasses (Fig. S5, Table 1) are similar to previous studies with comparable placement of the controversial phytoliths (Prasad *et al.* 2011; Christin *et al.* 2014; Burke *et al.* 2016). Our estimate for the node age of crown Chloridoideae (41.7 Ma; used for a secondary calibration in analysis of dataset 2) is slightly older than previous estimates but is within the 95% highest posterior density (HPD) interval of those studies (Table 1). Alternative placement of the controversial phytoliths at stem BOP+PACMAD led to estimates for crown Chloridoideae *c*. 10 Ma younger (32.1 Ma; Table S6).

The analysis focussing on Chloridoideae and *Triodia* chloroplast genomes allowed incorporation of non-coding regions into the alignment and produced a well-supported (posterior probabilities >0.99 for most nodes) chronogram for evolution in the group (Fig. 3). Estimated node ages (Table 2, rounded to two significant figures) indicate that *Triodia* began to diversify in the late Miocene (7.9 Ma; 7.0–8.8 Ma 95% HPD), and that the *T. basedowii* complex began to radiate in the Pleistocene (2.3 Ma; 1.9–2.7 Ma 95% HPD). Using a secondary calibration from the analysis with the alternate placement of the phytoliths at stem BOP+PACMAD had a small effect on the estimate for the crown age of the complex (1.7 Ma; 1.4–2.0 Ma 95% HPD; Table S6), but still indicated a radiation beginning in the Pleistocene.

As with a previous study on diversification timing for *Triodia* (Toon *et al.* 2015), the UCLN clock model produced younger estimates for node ages compared to the preferred RLC model (see Table 2). Restricting the alignment to *matK* resulted in older crown age estimates (Table 2) for *Triodia* (10 Ma) and the *T. basedowii* complex (3.8 Ma) compared to the full alignment; however, the estimates were still younger than those in Toon *et al.* (2015).

Our estimate for the timing of diversification should be interpreted cautiously, given our limited sampling of *Triodia* and the lack of a fossil calibration for our ingroup. Indeed, our estimate for the age of crown *Triodia* (7.0–8.8 Ma) is significantly younger than the 11.4–18.3 Ma estimate obtained in a recent study (Toon *et al.* 2015), although our estimate for stem *Triodia* is similar (see Table 2). This is surprising, especially given Toon *et al*. (2015) used a younger secondary calibration (*c*. 32 Ma) for crown Chloridoideae. There are several potential reasons for our dates being younger. First, we used a larger dataset (chloroplast genomic alignment *vs*. ITS + *matK*), which we expect to be more informative for inferring rates of evolution. When we used the *matK* region alone, we obtained an older estimate for crown *Triodia* (5.5–17 Ma), although still not as old as that of Toon *et al*. (2015). Second, our sampling of *Triodia* and outgroup Chloridoideae is sparser, which may have produced a node density effect (see Heath *et al.* 2008; Simon Ho, pers. comm.), limiting the number of inferred substitutions. While undersampling across a tree has been shown to result in younger ages, undersampling of a specific clade was not observed to have the same effect on the age of the subtending node (Linder *et al.* 2005), suggesting that undersampling in *Triodia* may not fully explain the discrepancy. Third, our sampling of *Triodia* lacks species from northern tropical regions, which were included in Toon *et al*. (2015) and which may have higher rates of evolution and could bias lower nodes to be older (see Beaulieu *et al.* 2015). Broader sampling of *Triodia* chloroplast genomes in the future may help to resolve whether our younger dates are primarily a result of limited sampling or a better estimate based on greater sequence data.

Table S4.

Partitions and models for datasets 1 and 2. The number of loci refers to genes, introns or intergenic spacers and is the number of possible partitions that PartitionFinder assigned to that partition.

**Dataset**	**Partition**	**Length (bp)**	**# loci**	**Model**
1	1	8103	10	GTR+I+G
	2	7146	13	GTR+I+G
	3	9049	9	GTR+I+G
	4	8235	14	GTR+I+G
	5	6600	12	GTR+I+G
	6	1543	1	GTR+G
	7	4780	3	GTR+I+G
	8	5390	6	GTR+I+G
	9	5924	6	GTR+I+G
	10	4605	4	HKY+I
	11	630	1	K80+G
2	1	13659	24	GTR+G
	2	21270	26	GTR+I+G
	3	13859	39	GTR+I+G
	4	7625	8	GTR+I+G
	5	6643	6	HKY+I+G
	6	1558	5	HKY+I+G
	7	7847	14	GTR+G
	8	7570	15	HKY+G
	9	3433	6	F81+I
	10	1021	1	HKY
	11	6563	8	GTR+G
	12	5849	6	HKY+I+G
	13	2888	1	HKY+I
	14	1725	4	K80+I

Table S5.

Molecular dating calibrations and their parameterization in BEAST. In the case of the secondary calibration, the age range is the 95% highest posterior density (HPD) interval, with the gamma implementation that closely approximates that HPD. The secondary calibration (S) is obtained from the analysis of dataset 1.

**Calibration**	**Type**	**Tree placement**	**Age (Ma) (95% quantile)**	**Distrib.**	**offset**	**mean**	**std dev**
1	fossil	stem Oryzeae	65 (–97.5)	log-normal	65	1	1.508
2	fossil	stem Pooideae	40 (–60)	log-normal	40	1	1.212
3	fossil	common ancestor of Stipa and Poa	34 (–51)	log-normal	34	1	1.115
4	fossil	crown Bambusoideae†	35 (–52.5)	log-normal	35	1	1.131
5	fossil	stem Chloridoideae	19 (–50)	log-normal	19	1	1.48
6	root bounding	root (crown Poaceae)	0 (–125)	uniform	0	-	-
S	secondary	crown Chloridoideae	41.7 (38.1–45.7)	gamma (41.7; 38.4–45.6)	36.1	7 (shape)	0.8 (scale)

Footnotes: References for fossils are: (1) (Prasad *et al.* 2005, 2011), (2) (Zucol *et al.* 2010), (3) (MacGinitie 1953; Manchester 2001), and (4) & (5) (Strömberg 2005). †(Piperno and Pearsall 1998).

Table S6.

Node ages (Ma) from analyses of datasets 1 (clear) and 2 (greyed) with the phytoliths placed alternatively at stem BOP+PACMAD or at stem Oryzeae (preferred). HPD is the highest posterior density interval.

**Analysis**	**Phytoliths at stem BOP+PACMAD**		**Phytoliths at stem Oryzeae**	
**Node**	**Age**	**95% HPD**	**Age**	**95% HPD**
crown Poaceae	110	96.6–124	123	119–125
crown BOP+PACMAD	62.5	59.6–66.5	82.4	78.5–86.5
crown Oryzoideae	44.4	39.8–49.2	65.6	65–66.6
crown Bambusoideae	38.6	35.1–43.3	50	40.9–59
crown Pooideae	47.2	44.1–50.4	60.1	55.1–65.2
crown Chloridoideae	32.1	29–35.1	41.7	38.1–45.7
stem *Triodia*	15.2	13.6–16.8	20.2	18.4–22.2
crown *Triodia*	5.92	5.19–6.65	7.89	6.98–8.82
crown *T. basedowii* complex	1.73	1.44–2.02	2.29	1.91–2.70

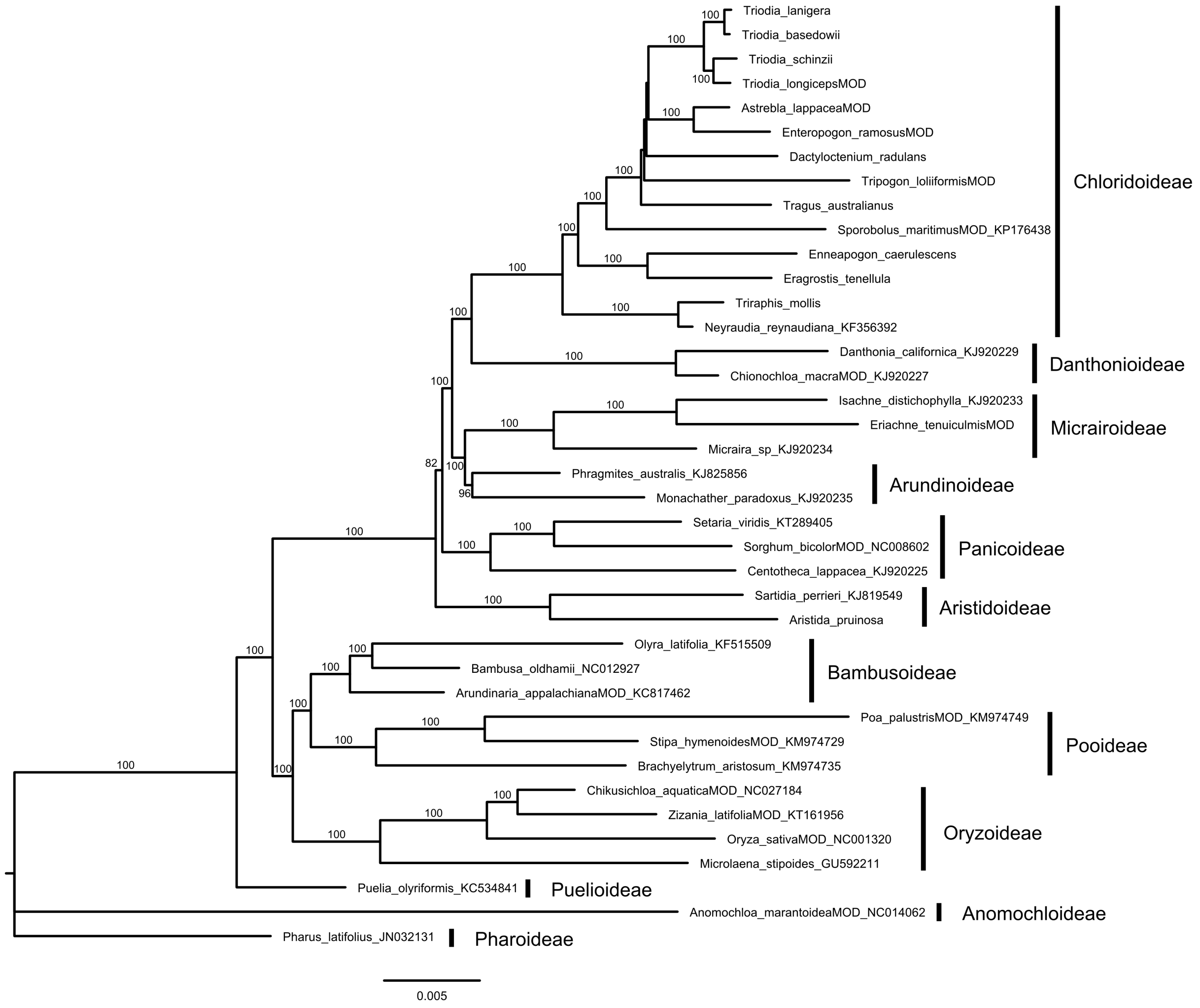

Figure S1.

RAxML tree for analysis of dataset 1. Alignment of 62,005 bp of chloroplast coding regions across Poaceae. Branch support from 100 rapid bootstrap replicates shown for values > 80. Grass subfamilies are indicated. Scale bar units are branch lengths from RAxML (inferred substitutions per site).

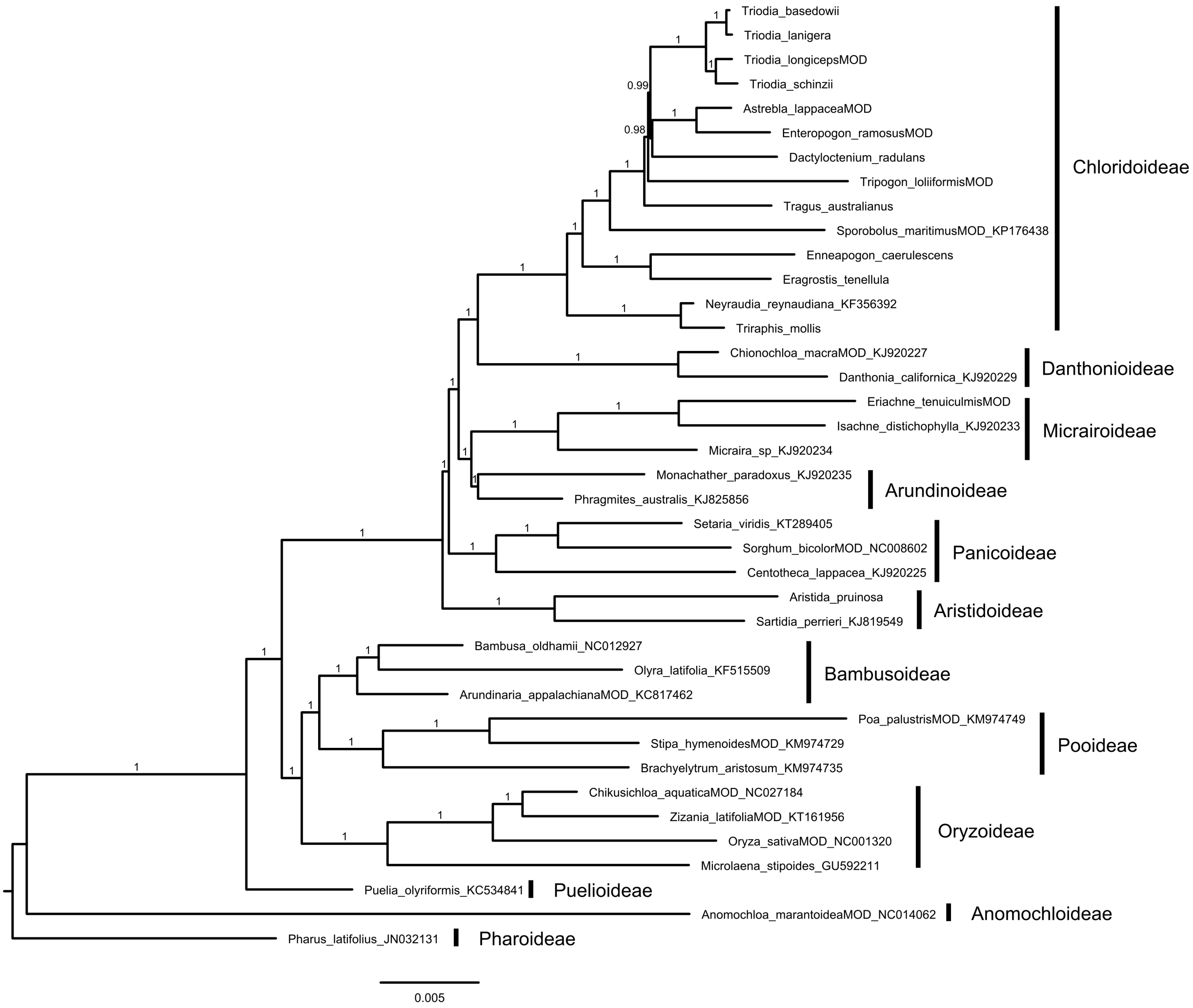

Figure S2.

MrBayes tree for analysis of dataset 1. Alignment of 62,005 bp of chloroplast coding regions across the Poaceae. Branch support values are posterior probabilities and are shown for values > 0.95. Grass subfamilies are indicated. Scale bar units are branch lengths from MrBayes (inferred substitutions per site).

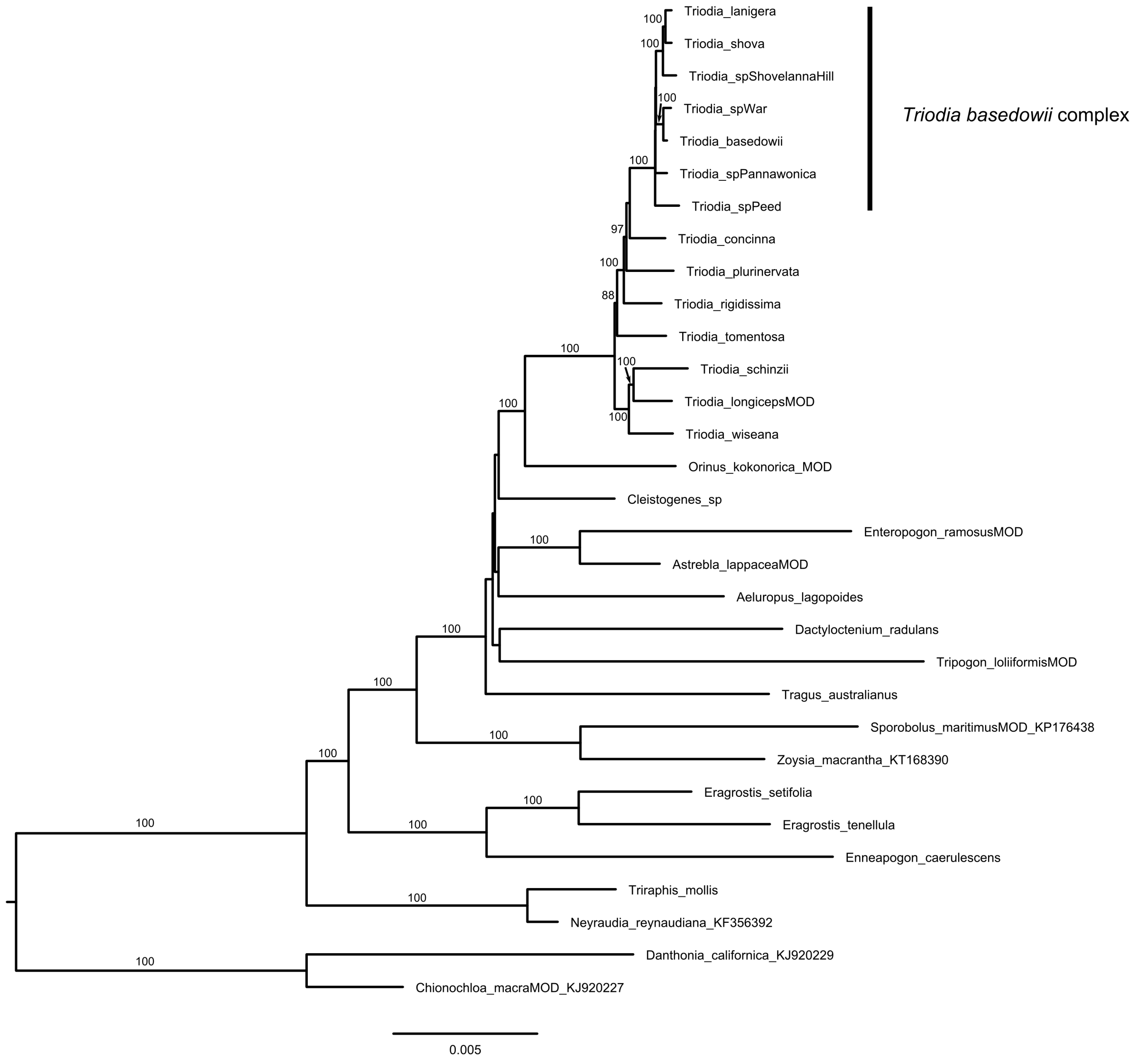

Figure S3.

RAxML tree for analysis of dataset 2. Alignment of 101,510 bp of chloroplast coding regions, spacers and introns for Chloridoideae with two outgroup Danthonioideae. Branch support from 100 rapid bootstrap replicates shown for values > 80. The *Triodia basedowii* complex is indicated. Scale bar units are branch lengths from RAxML (inferred substitutions per site).

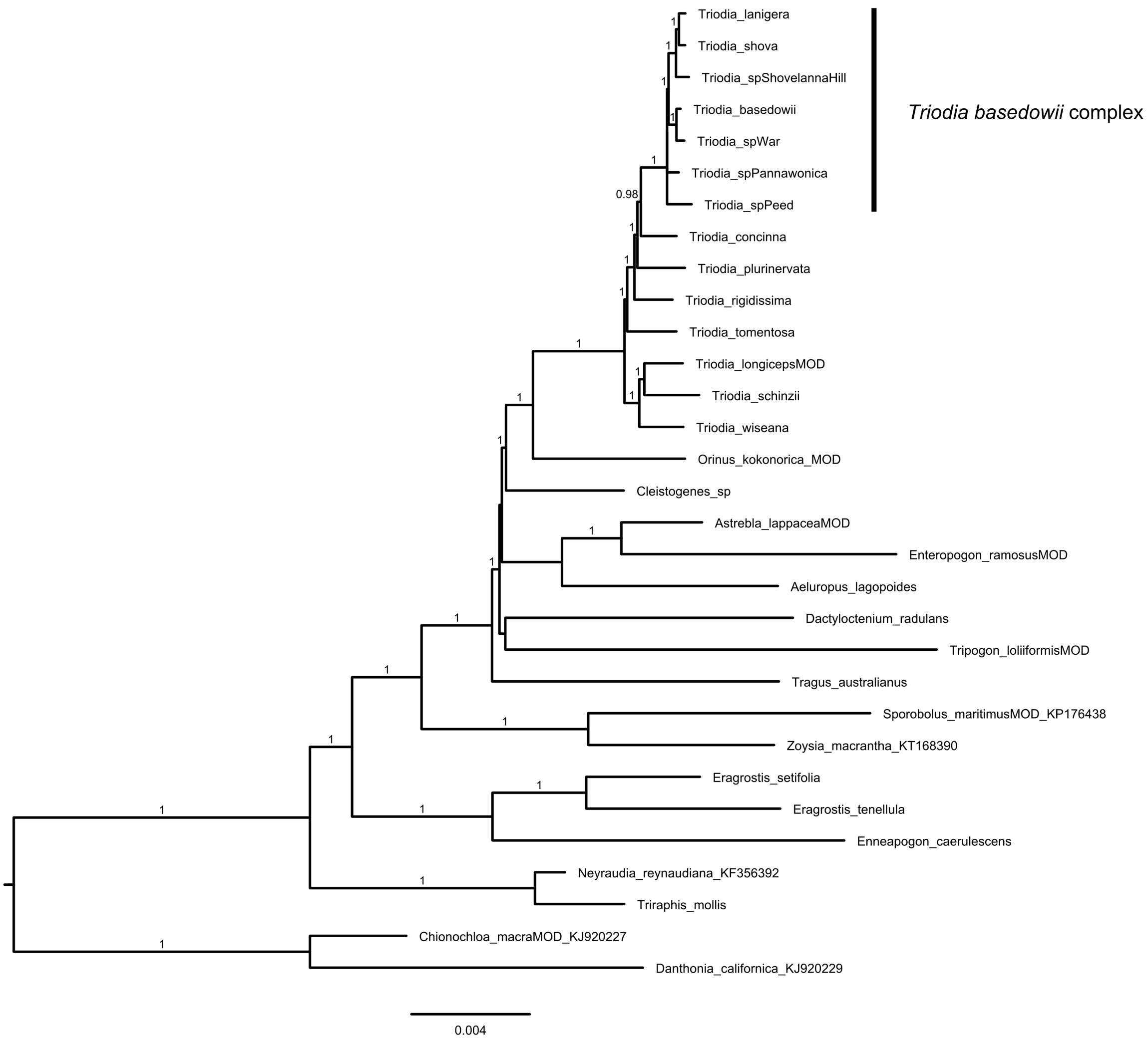

Figure S4.

MrBayes tree for analysis of dataset 2. Alignment of 101,510 bp of chloroplast coding regions, spacers and introns for Chloridoideae with two outgroup Danthonioideae. Branch support values are posterior probabilities and are shown for values > 0.95. The *Triodia basedowii* complex is indicated. Scale bar units are branch lengths from MrBayes (inferred substitutions per site).

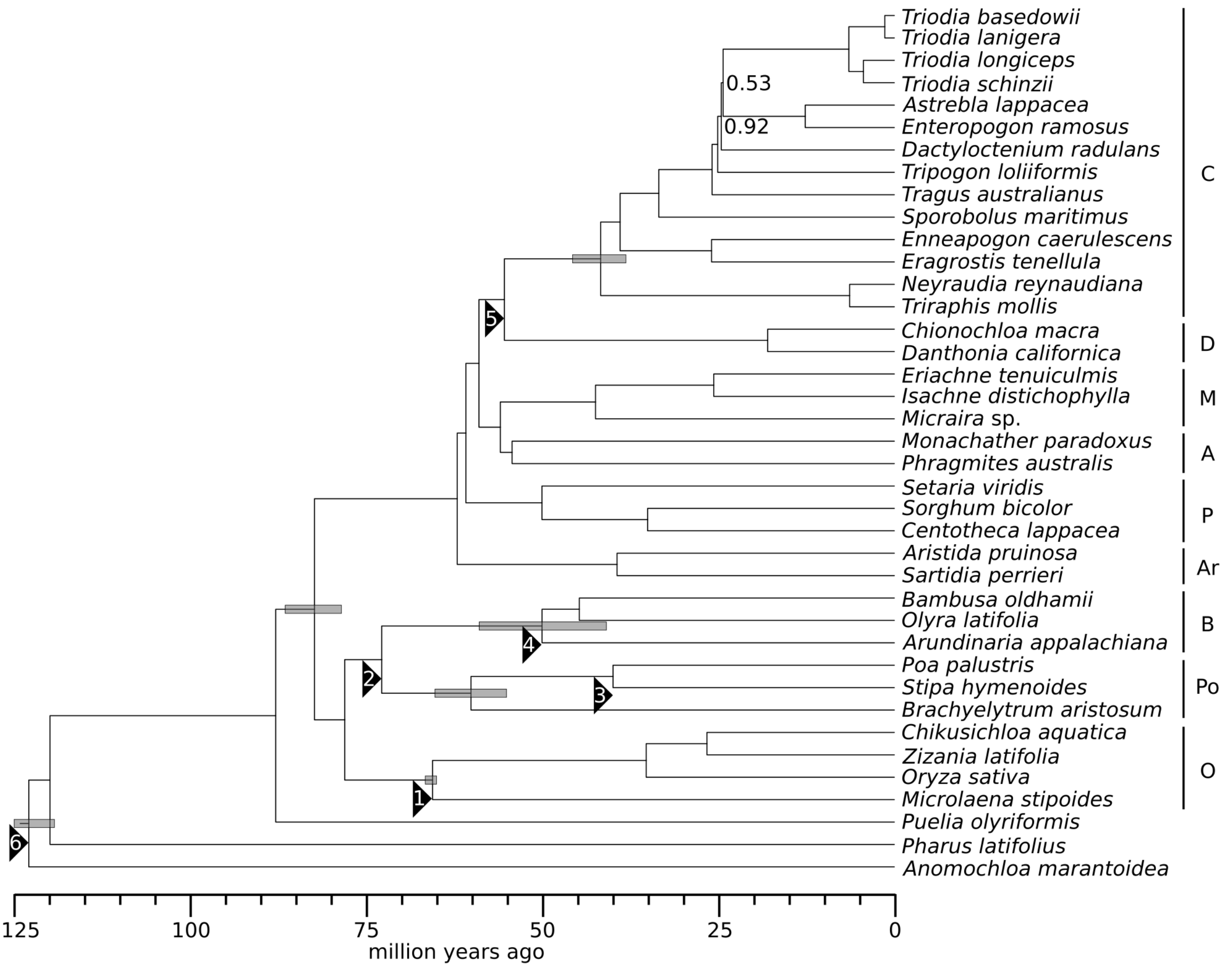

Figure S5.

Chronogram from the BEAST analysis of dataset 1, comprising chloroplast alignments from members of all grass subfamilies. Fossil calibration points are shown with black triangles and numbered as in Table S5. Node bars for selected nodes of interest are 95% highest posterior density intervals. Node support values are posterior probabilities and are shown for values < 1.00. Grass subfamilies in BOP+PACMAD are indicated at right; O: Oryzoideae, Po: Pooideae, B: Bambusoideae, Ar: Aristidoideae, P: Panicoideae, A: Arundinoideae, M: Micrairoideae, D: Danthonioideae, C: Chloridoideae.

SUPPORTING INFORMATION LITERATURE CITED

Anderson BM, Barrett MD, Krauss SL, Thiele K. 2016. Untangling a species complex of arid zone grasses (*Triodia*) reveals patterns congruent with co-occurring animals. *Molecular Phylogenetics and Evolution* **101**: 142–162.

Bankevich A, Nurk S, Antipov D, *et al.* 2012. SPAdes: a new genome assembly algorithm and its applications to single-cell sequencing. *Journal of Computational Biology* **19**: 455–477.

Beaulieu JM, O'Meara BC, Crane P, Donoghue MJ. 2015. Heterogeneous rates of molecular evolution and diversification could explain the Triassic age estimate for angiosperms. *Systematic Biology* **64**: 869–878.

Bouchenak-Khelladi Y, Salamin N, Savolainen V, *et al.* 2008. Large multi-gene phylogenetic trees of the grasses (Poaceae): progress towards complete tribal and generic level sampling. *Molecular Phylogenetics and Evolution* **47**: 488–505.

Bouckaert RR, Drummond AJ. 2017. bModelTest: Bayesian phylogenetic site model averaging and model comparison. *BMC Evolutionary Biology* **17**: 42.

Bouckaert R, Heled J, Kühnert D, *et al.* 2014. BEAST 2: a software platform for Bayesian evolutionary analysis. *PLoS Computational Biology* **10**: e1003537–e1003537.

Burke SV, Lin C-S, Wysocki WP, Clark LG, Duvall MR. 2016. Phylogenomics and plastome evolution of tropical forest grasses (*Leptaspis*, *Streptochaeta*: Poaceae). *Frontiers in Plant Science* **7**: 1993.

Christin P-A, Spriggs E, Osborne CP, Strömberg CAE, Salamin N, Edwards EJ. 2014. Molecular dating, evolutionary rates, and the age of the grasses. *Systematic Biology* **63**: 153–165.

Crisp MD, Hardy NB, Cook LG. 2014. Clock model makes a large difference to age estimates of long-stemmed clades with no internal calibration: a test using Australian grasstrees. *BMC*

Evolutionary Biology **14**: 263.

Dierckxsens N, Mardulyn P, Smits G. **2017**. NOVOPlasty: de novo assembly of organelle genomes from whole genome data. *Nucleic Acids Research* **45**: e18.

Doyle JA. **1992**. Revised palynological correlations of the lower Potomac group (USA) and the cocobeach sequence of Gabon (Barremian-Aptian). *Cretaceous Research* **13**: 337–349.

Doyle JJ, Dickson EE. **1987**. Preservation of Plant Samples for DNA Restriction Endonuclease Analysis. *Taxon* **36**: 715–722.

Drummond AJ, Ho SYW, Phillips MJ, Rambaut A. **2006**. Relaxed phylogenetics and dating with confidence. *PLoS Biology* **4**: e88.

Drummond AJ, Suchard MA. **2010**. Bayesian random local clocks, or one rate to rule them all. *BMC Biology* **8**: 114.

Duvall MR, Fisher AE, Travis Columbus J, *et al.* **2016**. Phylogenomics and plastome evolution of the chloridoid grasses (Chloridoideae: Poaceae). *International Journal of Plant Sciences* **177**: 235–246.

Gardner MG, Fitch AJ, Bertozzi T, Lowe AJ. **2011**. Rise of the machines--recommendations for ecologists when using next generation sequencing for microsatellite development. *Molecular Ecology Resources* **11**: 1093–1101.

Grass Phylogeny Working Group II. **2012**. New grass phylogeny resolves deep evolutionary relationships and discovers C_4 origins. *The New Phytologist* **193**: 304–312.

Heath TA, Hedtke SM, Hillis DM. **2008**. Taxon sampling and the accuracy of phylogenetic analyses. *Journal of Systematics and Evolution* **46**: 239–257.

Katoh K, Standley DM. **2013**. MAFFT Multiple Sequence Alignment Software Version 7: Improvements in Performance and Usability. *Molecular Biology and Evolution* **30**: 772–780.

Kearse M, Moir R, Wilson A, *et al.* 2012. Geneious Basic: an integrated and extendable desktop software platform for the organization and analysis of sequence data. *Bioinformatics* **28**: 1647–1649.

Kurtz S, Phillippy A, Delcher AL, *et al.* 2004. Versatile and open software for comparing large genomes. *Genome Biology* **5**: R12–R12.

Lanfear R, Calcott B, Ho SYW, Guindon S. 2012. Partitionfinder: combined selection of partitioning schemes and substitution models for phylogenetic analyses. *Molecular Biology and Evolution* **29**: 1695–1701.

Li H, Durbin R. 2009. Fast and accurate short read alignment with Burrows-Wheeler transform. *Bioinformatics* **25**: 1754–1760.

Linder HP, Hardy CR, Rutschmann F. 2005. Taxon sampling effects in molecular clock dating: an example from the African Restionaceae. *Molecular Phylogenetics and Evolution* **35**: 569–582.

MacGinitie HD. 1953. Fossil plants of the Florissant beds, Colorado. *Publications of the Carnegie Institution of Washington* **599**.

Manchester SR. 2001. Update on the megafossil flora of Florissant, Colorado. *Proceedings of the Denver Museum of Nature & Science* **4**: 137–161.

Martin M. 2011. Cutadapt removes adapter sequences from high-throughput sequencing reads. *EMBnet.journal* **17**: 10–12.

Piperno DR, Pearsall DM. 1998. The silica bodies of tropical American grasses: morphology, taxonomy, and implications for grass systematics and fossil phytolith identification. *Smithsonian Contributions to Botany* **85**: 1–40.

Poinar G, Alderman S, Wunderlich J. 2015. One hundred million year old ergot: psychotropic compounds in the Cretaceous? *Paleodiversity* **8**: 13–19.

Prasad V, Strömberg CAE, Alimohammadian H, Sahni A. 2005. Dinosaur coprolites and the early

evolution of grasses and grazers. *Science* **310**: 1177–1180.

Prasad V, Strömberg CAE, Leaché AD, *et al.* 2011. Late Cretaceous origin of the rice tribe provides evidence for early diversification in Poaceae. *Nature Communications* **2**: 480.

Python Software Foundation. **2016**. Python Language Reference, version 2.7.

Rambaut A. **2016**. *FigTree*. http://tree.bio.ed.ac.uk/software/figtree/.

Rambaut A, Suchard M, Xie D, Drummond A. **2014**. *Tracer*. http://tree.bio.ed.ac.uk/software/tracer/.

Ronquist F, Teslenko M, van der Mark P, *et al.* 2012. MrBayes 3.2: efficient Bayesian phylogenetic inference and model choice across a large model space. *Systematic Biology* **61**: 539–542.

Soreng RJ, Peterson PM, Romaschenko K, *et al.* 2015. A worldwide phylogenetic classification of the Poaceae (Gramineae). *Journal of Systematics and Evolution* **53**: 117–137.

Stamatakis A. **2014**. RAxML version 8: a tool for phylogenetic analysis and post-analysis of large phylogenies. *Bioinformatics* **30**: 1312–1313.

Stamatakis A, Hoover P, Rougemont J. **2008**. A rapid bootstrap algorithm for the RAxML web servers. *Systematic Biology* **57**: 758–771.

Strömberg CAE. **2005**. Decoupled taxonomic radiation and ecological expansion of open-habitat grasses in the Cenozoic of North America. *Proceedings of the National Academy of Sciences of the United States of America* **102**: 11980–11984.

Toon A, Crisp MD, Gamage H, *et al.* 2015. Key innovation or adaptive change? A test of leaf traits using Triodiinae in Australia. *Scientific Reports* **5**: 12398.

Urban MA, Nelson DM, Jiménez-Moreno G, Châteauneuf J-J, Pearson A, Hu FS. **2010**. Isotopic evidence of C_4 grasses in southwestern Europe during the Early Oligocene–Middle Miocene. *Geology*

38: 1091–1094.

Walker BJ, Abeel T, Shea T, *et al.* 2014. Pilon: an integrated tool for comprehensive microbial variant detection and genome assembly improvement. *PLoS ONE* **9**: e112963.

Wysocki WP, Clark LG, Kelchner SA, *et al.* 2014. A multi-step comparison of short-read full plastome sequence assembly methods in grasses. *Taxon* **63**: 899–910.

Zerbino DR, Birney E. **2008**. Velvet: Algorithms for de novo short read assembly using de Bruijn graphs. *Genome Research* **18**: 821–829.

Zucol AF, Brea M, Bellosi ES. **2010**. Phytolith studies in Gran Barranca (central Patagonia, Argentina): the middle-late Eocene In: Madden RH, Carlini AA, Vucetich MG, Kay RF, eds. *The Paleontology of Gran Barranca: Evolution and Environmental Change through the Middle Cenozoic of Patagonia*. New York: Cambridge University Press, 317–340.
